# Genie: an interactive real-time simulation for teaching genetic drift

**DOI:** 10.1186/s12052-022-00161-7

**Published:** 2022-02-21

**Authors:** Andreina I. Castillo, Ben H. Roos, Michael S. Rosenberg, Reed A. Cartwright, Melissa A. Wilson

**Affiliations:** 1 Department of Environmental Science, Policy and Management, University of California, Berkeley, USA.; 2 The Biodesign Institute, Arizona State University, Tempe, USA.; 3 Center for Biological Data Science, Virginia Commonwealth University, Richmond, USA.; 4 School of Life Sciences, Arizona State University, Tempe, USA.; 5 Center for Evolution and Medicine, Arizona State University, Tempe, USA.

**Keywords:** Genetic drift, Simulations, Evolution, Education

## Abstract

Neutral evolution is a fundamental concept in evolutionary biology but teaching this and other non-adaptive concepts is especially challenging. Here we present Genie, a browser-based educational tool that demonstrates population-genetic concepts such as genetic drift, population isolation, gene flow, and genetic mutation. Because it does not need to be downloaded and installed, Genie can scale to large groups of students and is useful for both in-person and online instruction. Genie was used to teach genetic drift to Evolution students at Arizona State University during Spring 2016 and Spring 2017. The effectiveness of Genie to teach key genetic drift concepts and misconceptions was assessed with the Genetic Drift Inventory developed by Price et al. (CBE Life Sci Educ 13(1):65–75, 2014). Overall, Genie performed comparably to that of traditional static methods across all evaluated classes. We have empirically demonstrated that Genie can be successfully integrated with traditional instruction to reduce misconceptions about genetic drift.

## Introduction

A well-recognized challenge in biological science education is successfully teaching evolutionary concepts (Alters and Nelson 2002). Within this discipline, some topics remain more challenging to teach than others, and the number and efficacy of tools available for instruction varies (Shulman 1987; Ziadie and Andrews 2018). For instance, multiple strategies have been developed to improve the teaching of concepts like natural selection (Ziadie and Andrews 2018) while the best practices for teaching equally important topics such as non-adaptive evolution remain largely understudied (Kalinowski et al. 2013). This is particularly problematic for topics like genetic drift because concepts of adaptive and non-adaptive evolution form independent elements in evolutionary thinking, and a better understanding of one does not necessarily imply a better comprehension of the other (Beggrow and Nehm 2012). To address this, studies devoted to developing, improving, and testing teaching strategies for non-adaptive evolutionary concepts are needed.

Previous studies have created approaches aimed to identify student misconceptions regarding genetic drift (Andrews et al. 2012; Price et al. 2014); in addition, study activities and software have been developed, tested, and made publicly available (Price et al. 2016; Revell 2019; Staub 2002). These serve as indicators that the knowledge gap regarding genetic drift instruction is being addressed. Nonetheless, diverse class environments, student cohorts, and even teaching styles require distinct sets of tools. Furthermore, there is an academic push for improving the teaching strategies currently set in place and to utilize alternative instruction methods (Lee et al. 2017; Nelson 2008; Tanner and Allen 2005). In fact, more holistic strategies such as transformative teaching—centered around helping students master key course concepts as well as developing learning-related values and skills—have been proposed (Slavich and Zimbardo 2012). Similarly, teaching strategies that favor discussing and testing evolutionary concepts among students have been shown to be effective (Nehm and Reilly 2007). As a result, tools that can be used to facilitate free in-class exploration of evolutionary concepts are especially useful since they allow students to both learn these concepts and develop critical thinking skills.

Here, we developed Genie, a web application designed to demonstrate several population genetics and evolutionary notions including genetic drift, gene flow, and random mutation. Genie simulates evolution in real time in a finite population using cellular automata. Our intuition is that spatially explicit, individual-based simulations allow students to visualize genetic drift in a way not available from line graphs of allele frequency changes, including how changes at the individual level of the population translate into fluctuations in allele frequency and fixation or loss of alleles. This web-based software is accessible to students and leads to increased knowledge of geneticdrift concepts, as tested using a Genetic Drift Inventory (Price et al. 2014). These types of assessments have proven to be useful in capturing students’ understanding of other complex evolutionary concepts in the past (Perez et al. 2013). Genie requires no setup other than navigating to a web page, thus making the use of stochastic simulations to demonstrate genetic drift practical to both educators and students.

## Methods

### Genie simulation program

Genie (https://cartwrig.ht/apps/genie/) is a web-based, stochastic simulation app written in JavaScript. The simulation uses a spatially explicit Moran model (Nei et al. 1976) to describe a finite population of 1,024 individuals on a 32 by 32 grid. Individuals are haploid with a single locus. The locus mutates according to the infinite alleles model (Nei et al. 1976). Genie works as follows:

### Population initialization

The simulation begins when a population is randomly initialized according to Hoppe’s Urn (Perez et al. 2013). Briefly, the population is created one individual at a time, and each individual either carries a new, unique allele or is a copy of a previously created individual. The probability that individual *i* + 1 has a new allele is *θ/(θ*+*i)* and the probability that the individual copies an existing allele is *(i)/(θ*+*i)*, where *θ* = *2Nμ*, *N* is the population size, and *μ* is the mutation rate. If an individual copies an allele, it randomly chooses a previously initialized individual uniformly. At initialization *μ* is = *0.001* to ensure diversity within the initial population, but the mutation rate of each generation can be specified by the user, defaulting to 0.

### Algorithm

At each step of the simulation, a randomly selected individual dies, leaving its corresponding cell momentarily empty. A parent allele is then randomly selected from the eight immediate neighboring cells (including adjacent and diagonal). Cells on the edges and corners of the simulation have fewer neighbors than internal cells, causing a small edge effect. The probability that a new individual will have the same allele as its parent is 1-*μ*, and the probability that an individual has a new, unique allele is *μ*. Each ‘generation’ consists of 2000 death/birth steps after which the population is redrawn in the visualization window.

### Running

The application contains four components: a grid, where the population is displayed (Additional file 1a); a control panel, where users can manipulate the simulation’s mutation parameter (Additional file 1b); an upper graph, where users can see the number of alleles in the population at any given time (Additional file 1c); and a lower graph, where users can see the frequency of different alleles at any given time (Additional file 1d). Both graphs update in real time as the simulation runs. Each initial allele is assigned one of 18 basic colors, while each mutant allele is assigned one of six neon colors. A single button allows users to toggle between starting the simulation or pausing it. A reset button allows users to restart and reinitialize the simulation at any point.

### Barriers

Users can create a barrier in the population grid. To do so, users alter a cell by clicking on it or alter a set of cells by clicking and dragging the cursor to select multiple cells. When a barrier is created, the color associated with the cell changes to black. Barriers act neither as parent cells (they are never replicated) nor die. Thus, for each created barrier cell the total population size declines by one. By building barriers, users can construct physical constraints that restrict the movement of alleles between subpopulations. Barriers can be used to create subpopulations of different sizes and shapes, as well as to study the effects of corridors on gene flow. Barriers can be removed by clicking on the chosen cell(s) a second time; this will set the cell color to white and designate the cell as unoccupied. Neighboring cells will replicate into unoccupied cells; unoccupied cells cannot serve as a parent of a neighboring cell.

### Forced mutation

Users can force a mutation to occur in a manner similar to creating barriers. Cells can be mutated by holding the SHIFT button while clicking the cell, or while clicking and dragging the cursor across several cells. Forcing a mutation immediately creates a new, unique allele in each of the chosen cell(s).

### Instruction

Before each recitation section, all participants received a lecture on genetic drift. Following, all recitation classes received further instruction of genetic drift by their corresponding Graduate Teaching Assistants (TAs). For the participants in the Non-Genie 2017 class (control), the TAs used static images in a worksheet, taken from screenshots of Genie, to explain genetic drift. These images were comparable to textbook images of differences in allele frequencies. For the participants in the Genie 2016 and Genie 2017 classes, the basic features, display, and usability of the Genie software were explained, and questions designed to facilitate discussion and interpretation were provided. The recitation slides (Additional file 2) were made available to all students after all recitation sessions concluded. Four main activities were conducted:

#### Activity 1: Defaults parameters/settings

Participants ran Genie without modifying any parameters or creating barriers. As the number of generations increased, participants kept track of the changes in the number of alleles in the population and the allele frequencies. Participants made conjectures on the distribution of haplotypes in the population by tracking variations in the colors patterns (alleles) shown in the population grid. The mutation rate was not modified; however, participants recorded new alleles arising at any point of the simulation. The simulation ran until one allele reached fixation, and participants kept track of the number of generations until this occurred.

#### Activity 2: Effects of absolute barriers on genetic drift and gene flow

The simulation was re started and participants created two barriers reaching opposite borders of the population grid (one horizontal and one vertical). This setup resulted in four completely isolated populations of roughly equal size. No modifications in the mutation rate were introduced. Participants kept track of variations in alleles, changes in number of alleles, and allele frequency. Additionally, participants recorded the allele number and distribution in each of the four independent sections/populations. The simulation continued until one allele became fixed in each section/population. After one allele became fixed in each section/population, participants paused the simulation and created a corridor by removing part of a barrier. Participants recorded the changes in number of alleles and allele frequency, as well as the movement of alleles between connected sections/populations. The simulation ran until one allele became fixed. The number of generations for an allele to become fixed amongst independent sections/populations was recorded.

#### Activity 3: Effects of partial barriers and corridors on genetic drift and gene flow

Participants restarted the simulation and created barriers that entirely separated the population grid into four sections of roughly equal size. Before the simulation started, participants formed a corridor by removing a portion of the barriers. This setting allowed for gene flow to occur between sections/populations from the beginning of the simulation. Participants recorded the changes in number and allele frequency between (1) completely isolated sections/populations; and (2) sections/populations connected by the corridor. Participants compared the flow of alleles across the corridor with that observed in Activity 2. Additionally, participants also recorded the number of generations until fixation was reached in connected and isolated areas. The mutation rate was not modified in this activity.

#### Activity 4: Effects of mutation rate on genetic drift

Participants restarted the simulation, increased the mutation rate, and recorded the changes in the population grid and accompanying graphs. Participants repeated the activity reducing the mutation rate. No barriers were created on the population grid.

After the four main activities were completed, participants were allowed to freely explore other potential outcomes of genetic drift. Participants freely modified the population landscape by creating various types of barriers and/or changing the mutation rate. Participants followed series of suggestions activities/questions: Evaluate the effects of creating barriers of different size and shape; assess the effects of genetic drift on different population sizes; discern the effects of genetic drift on allele diversity within a single population, and between isolated populations; observe the effects that creating corridors with different size and shapes have on gene flow; evaluate the effects of creating corridors and barriers at different points of the simulation; and track the effects of modifying the mutation rate at different points of the simulation.

### Data collection

Genie’s efficacy as a tool for teaching Genetic Drift concepts was tested in the Evolution (BIO345) class at Arizona State University (ASU). Genie was used during the practical portion (recitation) of the BIO345 course in the Spring 2016 and Spring 2017 classes. All participants in the Spring 2016 class used Genie during practical class sessions. In the Spring 2017 class, half of the participants used the dynamic visualization of Genie while the other half used static illustrations. Participants in both the Spring 2016 and Spring 2017 classes were given the option to opt-in to the study at the end of the semester. In addition, participants were given the option to provide their demographic information: reported gender, reported ethnicity, and first-generation college student status. All research was reviewed and approved by Arizona State University’s IRB protocol STUDY00003707.

The impact of Genie as a tool for teaching concepts of genetic drift was evaluated using the Genetic Drift Inventory (Price et al. 2014). The inventory was used without changes (22 questions assessing different aspects of genetic drift) in pre- and post-recitation assessments. The pre- and post-recitation assessments (considered as homework for the entire class) were individually answered by each participant. The pre-recitation assessment was posted online on Blackboard two days before recitation. Participants were asked to answer all questions by 3:00 pm on the day of the recitation. The post-recitation assessment was posted on Blackboard at 9:00 pm after the last recitation session ended. Participants had two days to individually complete the post-recitation assessment. All participants were allowed the same amount of time to complete both the pre- and post-recitation assessments. Participants’ answers were recorded, and their individual pre- and post-recitation scores were calculated by summing the number of correctly answered questions (value 1 point) out of the 22 questions in the Genetic Drift Inventory.

### Genie assessment

The complete dataset was divided into two major groups based on the instruction year. These groups were: the entire Spring 2016 class (henceforth referred to as Genie 2016) and the entire Spring 2017 class. The 2017 class was further subdivided into groups based on the instruction method used during the practical class session. These groups were: participants that used Genie during the recitation session in 2017 (henceforth referred to as Genie 2017) and the participants who did not use Genie during the recitation session in 2017 (henceforth referred to as Non-Genie 2017). The Genie 2016 class was subsequently divided into eight in-class groups of roughly equal size, while each 2017 class was divided into four in-class groups of roughly equal size (two Genie and two Non-Genie). The groups were designated based on recitation start times, TA pairs; and in the case of 2017, on the use of dynamic (Genie) vs. static (Non-Genie) instruction methods. No more than 48 participants participated in each recitation session. All analyses and figures were developed using R v3.2. The code and datasets used are available (Additional files 3, 4, 5, 6, 7, 8, 9, 10, 11, 12, 13, https://github.com/AndreinaCastillo/Genie_manuscript_data_analysis).

The putative relationship between participants’ demographics and the pre- and post-recitation scores was evaluated using a two-way ANOVA. The following demographic parameters were used as explanatory variables: reported gender, reported ethnicity, and first-generation college student status. In the case of 2017, the use of Genie as an instruction tool was also considered as an explanatory variable. The two-way ANOVA was performed independently for Genie 2016, Genie 2017, and Non-Genie 2017. Next, we assessed if the pre- and post-recitation performance varied between the three class groups or among subgroups within each class. To conduct this analysis, the distribution of pre- and post-recitation scores was assessed using the ‘fitdistrplus’ (Delignette-Muller and Dutang 2015) and ‘betareg’ (Cribari-Neto and Zeileis 2010) R packages. Potential differences between pre- and post-recitation scores were evaluated both between classes and within each in-class group. Cohen’s d was used to measure the effect size between pre- and post-recitation scores within each class, and to estimate differences in pre- and post- recitation scores between Genie 2017 and Non-Genie 2017. In addition, a paired Student’s t-test was performed between individual participants’ pre- and post-recitation scores within each class.

Finally, question-specific performance was evaluated to determine how Genie aided participants in addressing the specific genetic drift concepts and misconceptions listed in the Genetic Drift Inventory (Price et al. 2014). The number of correct answers in pre- and post-recitation sessions associated with each question were calculated from participants’ individual answers, and the totals were then compiled by class. Differences between pre- and post- recitation scores for each question were assessed using a McNemar’s χ^2^ test. In addition, the difference in the number of correct answers per question in Genie 2017 vs. Non-Genie 2017 pre- and post-recitation sessions was assessed using a Fisher’s exact test.

## Results

Demographic representation varied among cohorts (Table 1). A two-way ANOVA found that most demographic explanatory variables did not affect pre- and post-recitation scores (Table 2) with the exception of ‘First-generation’ college in post-recitation scores (F = 7.955, p-value = 0.005) for Genie 2016, and ‘Genie used’ in pre-recitation scores (F = 6.131, p-value = 0.014) for 2017. In the case of the ‘First-generation’ college students, lower pre-recitations scores were observed in ‘Not First-generation’ students from Genie 2016 and Genie 2017, while the opposite trend was observed in Non-Genie 2017. It should be noted that ‘First-generation’ college students showed slightly less improvement than ‘Not First-generation’ college students despite both groups having higher post- than pre-recitation scores. Overall, pre- and post-recitation scores were different among the classes analyzed. The mean pre- and post-recitation scores for Genie 2016 were lower than in either Genie 2017 or Non-Genie 2017. Differences in post-recitation scores could be largely explained by the initial class performance (Table 3). In-class groups showed similar performance levels in all evaluated groups except for ‘TA Pair1 7:30 pm’ (p-value = 0.017) during Genie 2016, this class was composed exclusively of honor students.

**Fig. 1:**
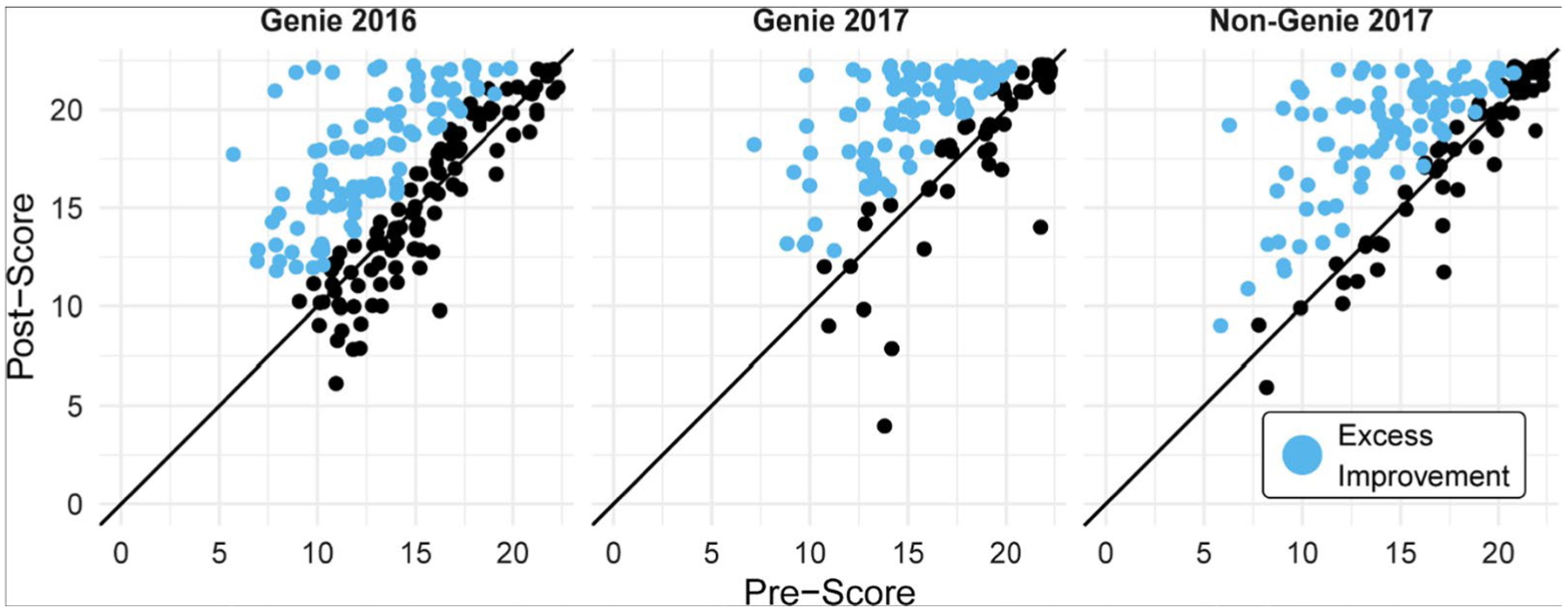
Students’ test scores improved after instruction. Blue dots represent excess improvement in class performance. The presence of blue points in a graph indicates that there were more students whose post-test score was better than their pre-test score. The number of blue points indicates how many more students improved their scores than students whose scores decreased

**Fig. 2:**
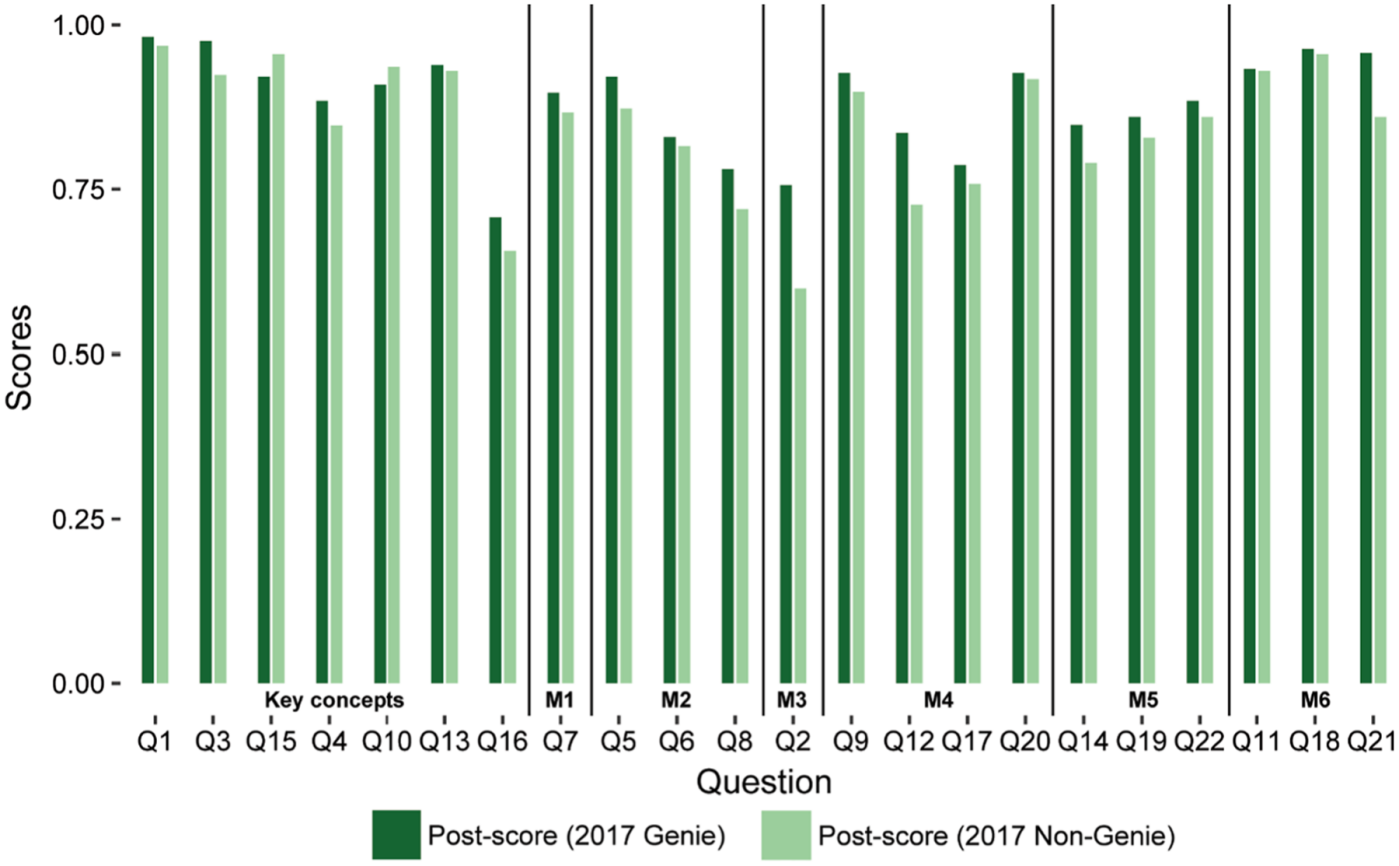
Post-recitation scores by question (Price et al. 2014) were generally higher in Genie 2017 compared to Non-Genie 2017. A bar plot comparing Genie 2017 (dark green) and Non-Genie 2017 (pale green) is shown. Questions have been grouped according to the classification provided by Price et al. (2014), with questions pertaining to Key concepts and misconceptions (M1-M6) separated by horizontal bars

Overall, comparisons of pre- and post-recitation scores showed that students performed better in all classes regardless of the instruction method used (Fig. 1). Cohen’s d values (Table 4) showed a moderate improvement in post-recitation scores compared to the pre-recitation scores in Genie 2016 (0.608, CI: 0.408–0.807), Genie 2017 (0.632, CI: 0.410–0.855), and Non-Genie 2017 (0.658, CI: 0.430–0.886). This was also true for most individual participant scores (Table 5, Additional file 14). Understanding of key genetic drift concepts and misconceptions statistically improved after instruction with or without Genie (Table 6). Post-recitation scores were generally higher in Genie 2017 than in Non-Genie 2017 except for two questions (Q10 and Q15, Fig. 2). Fisher’s exact test showed that the instruction method (Genie vs. Non-Genie) was not associated with student’s switching answers from correct to incorrect or incorrect to correct between pre- and post-recitation (Table 7). Results were comparable with or without including students within honor sections (Additional file 15).

## Discussion

There are numerous software options capable of generating genetic drift simulations. Some of them can be easily downloaded and installed (Kliman et al. 2008; Revell 2019), others include an ample array of parameters to be modified by the user (http://evolution.gs.washington.edu/popgen/popg.html), and others can be found publicly available online (e.g. the Genetic Drift Simulator (http://www.biology.arizona.edu/evolution/act/drift/drift.html or Phyletica (http://phyletica.org/teaching/drift-simulator/). Some of these software even have a dynamic interface similar to that developed by Genie (http://virtualbiologylab.org/NetWebHTML_FilesJan2016/RandomEffectsModel.html). While this list is not exhaustive, it provides a glimpse on how computational tools, and especially those found freely in web-interfaces, are becoming predominantly used in science teaching. The objective of this paper is not to compare Genie’s performance to all these tools, instead, the authors aim to present an additional teaching tool that can be added to an instructor’s repertoire. As such, we endeavor to show that Genie can be efficiently used alongside other class instruction methods. A comparison was made between Genie-based instruction and instruction using static images (henceforth referred as teacher-centered instruction). The comparison was chosen since teacher-centered methods still are commonly used in science teaching (Tanner and Allen 2004) and have been traditionally used when teaching evolutionary topics in ASU.

There were no significant differences in the performance levels among participants from distinct demographic backgrounds. Despite differences in levels of representation across groups, pre- and post- recitation scores were similar. However, while participant’s performance increased in all methods of instruction, ‘First-generation’ college students showed slightly lower improvement than ‘Non-first generation’ college students. Multiple studies have attempted to address the social class gap among undergraduate students and explain why ‘First-generation’ college students, on occasion, perform more poorly than ‘Non-first generation’ college students (Grineski et al. 2018; Tibbetts et al. 2018). One finding pertinent to our assessment is that ‘First-generation’ college students tend to underperform when they know that their performance is going to be compared to that of other students in the class (Jury et al. 2015). This might be an unintended consequence of the in-class methods used here, which favored in-class discussion and student participation. However, while not possible to address here, these results could point towards the unique disadvantages and social-related pressures that ‘First-generation’ college students face within ASU. These results should be evaluated in more detail in future studies.

Overall, participants’ performance was not affected by the instructor or the participant populations within the group, except for the ‘TA Pair1 7:30 pm’ group during Genie 2016. The ‘TA Pair1 7:30 pm’ group was formed by a small number of honors students; therefore, it is possible that this group performed better compared to the general class population in Genie 2016. Previous studies have found that instructors’ mastery of the content, as well as their overall teaching style play a critical role in students’ learning process (Alsharif and Qi 2014; Maleki et al. 2017). Thus, our results are indicative that Genie performs similarly well even with teachers using diverse teaching styles and having variable levels of expertise.

In that regard, we were unable to control for previous classes that BIO345 students took. Although, all students in BIO345 are required to have passed BIO340 (General Genetics), which typically includes instruction in evolutionary genetics; BIO340 is taught by multiple instructors, who do not teach evolutionary genetics equally. Interestingly, despite these differences, mean scores showed that the increase in performance between pre- and post-recitation was ~ 0.1 regardless of the teaching method used in BIO345. The main distinction were the pre-recitation scores, with some classes initially performing better than others. Pre-course/test assessments are used to contextualize post-course/test scores. Previous data shows that lower pre- scores often result in lower post- scores during midterms and finals (Furrow and Hsu 2019). Interestingly, the pre-recitation scores observed here were larger than in other studies using the Genetic Drift Inventory; in fact, the smallest pre-recitation score reported here (0.645 for Genie 2016) was higher than pre-recitation score reported during the assessment of The Genetic Drift and Bottlenecked Ferrets module (0.58) by Price (2016). This suggests that the starting performance level of the classes was higher than in other studies, which could be due to genetic concepts being introduced in lecture and before recitation. Taken together, these results are indicative that both teacher-centered and Genie-based teaching strategies led to a comparable improvement in participant’s scores, regardless of the initial performance level of the class. Thus, it is possible to conclude that Genie can perform as efficiently as traditionally teacher-centered instruction.

The lack of statistically significant differences between Genie-based and teacher-centered instruction also deserves some note. All students received the same lecture, were taught by the same professor, had access to the same set of slides, and followed the same worksheet instructions. The only difference was that participants in the Non-Genie 2017 class had static images in the worksheet, while participants in the Genie 2017 worked with the simulation software. Therefore, improvements in comprehension of genetic drift and related concepts may originate from any of those common factors and not from the use of Genie itself. Nonetheless, previous studies have demonstrated that cellular automata simulations can be effective tools for teaching evolution in action. In fact, the most well-known of these tools, Avida-ED, has been shown to serve multiple teaching purposes. First, digital simulations like Avida-ED or Genie provide an experimental platform in which students can test the effect of core evolutionary mechanisms; second, these simulations encourage students to use inquiry-based learning; and third, the simulations allow students to learn concepts in a manner that is transferable across levels of biological complexity (Bray Speth et al. 2008; Robert 2007; Smith et al. 2016). Simulations also provide multiple independent instances for students to observe the stochasticity in genetic drift, unlike a lesson that only walks through changes in allele frequencies. In this sense, the simulated individuals undergo real evolution, experiencing mutation, replication, selection, and drift. Avida-ED has detailed exercises and a lab manual describing genetic drift (i.e., https://avida-ed.msu.edu/files/curricula/LabBook/Avida-ED_LabBook_Ex4.pdf).

Overall, understanding of genetic drift key concepts and misconceptions improved following instruction with all teaching strategies. Previous analyses have shown that a combination of traditional teaching-centered methods, with student-centered methods, and active learning strategies results in superior student performance (Dolan and Collins 2015; Shir et al. 2016; Wieman 2014) and that concept inventories are an effective way to support and evaluate undergraduate learning of evolution concepts (Furrow and Hsu 2019). In particular, the Genetic Drift Inventory has been found to be a generally reliable tool for inferring knowledge changes on upper-level undergraduates (Tornabene et al. 2018). However, despite their advantages, previous research also suggests that concept inventories could be used more creatively. For instance, in the present study, the changes in participant’s performance might be related to students becoming familiar with the questions found in the Genetic Drift Inventory. Or in other words, to the study being linked to the exclusive use of the Genetic Drift Inventory as presented by the original authors. While this is undoubtedly a factor, we expect that all questions should be affected equally, which should not largely bias our results. Moreover, we observed that the changes in student performance varied among the Genetic Drift Inventory questions, suggesting that teaching strategies did have an impact on participants’ performance. Nonetheless, future uses of the Genetic Drift Inventory should be enriched by incorporating new multiple choice and open-ended questions in post-recitation assessments.

In the case of evolution teaching, strategies that favor student’s development of critical thinking skills are especially useful. For instance, tools and methods that aid in creating and testing hypotheses have been effective in improving students’ understanding and acceptance of evolutionary theory (Lark et al. 2018; Smith et al. 2016). Likewise, instruction using computer simulation programs has proven to be valuable in facilitating student’s recognition of the breadth of evolutionary mechanisms that can act in a population (Kliman 2001). Nonetheless, different students can master the same topic using different paths (Price et al. 2016), and different classroom settings might be more suitable for distinct teaching methods. Simply put, there is no ‘fit all’ teaching strategy that can be universally implemented. Therefore, providing instructors with a broad repertoire of teaching tools can aid them in finding those that better work for the topic being instructed, the specific class needs, and the instructor style. In this regard, we expect Genie can be added to the repertoire of higher education tools to be used for teaching genetic drift and other non-adaptive evolution concepts.

## Conclusion

The present study shows that Genie can be successfully used for teaching concepts related to genetic drift and non-adaptive evolution to undergraduate students. Genie performed comparably to traditional teacher-centered methods across all evaluated groups. Moreover, Genie-based and teacher-centered approaches led to participants understanding distinct key concepts and misconceptions of genetic drift. This indicates that Genie can be effectively used alongside other teaching strategies to provide a rounded view of non-adaptive evolution. In a related note, Genie provides a means for participants to develop and test their own hypotheses, which can be useful in practicing critical thinking skills. Despite this positive outcome, it should be noted that this manuscript only describes the first two instances in which Genie was used as a teaching tool in an undergraduate class, and therefore, there is room for improvement on the software’s implementation. For one, Genie could be presented to students before or during lecture as to determine how the software influences their understanding of genetic drift concepts when they are first introduced. In addition, recitations could allocate time for students to freely interact with the software at the start of the session, that way, students could infer what the different feature of the program do, how they affect the population, and how they could use them to test evolutionary hypotheses (this could even be done as a homework). Alternatively, questions allowing students to evaluate their understanding of genetic drift concepts in relation to the usefulness of the tool should be included in future classes. The effectiveness of Genie should also be further evaluated across institutions as well as in classes with higher pre- score performance than obtained here.

## Supplementary Material

SFile3_Genie **Additional file 3.** R code used to conduct the analyses in this study. Code to perform the analyses after removing honor students from the population is also included. Participant’s data was split into multiple files to facilitate code modularity. Additional files 4–13 represent the data used on the study by different segments of the R code.

Sfile4_BIO345_DEMOGRAPHICS_2016 **Additional file 4.** Text file containing demographic data of participants in the BIO345 Genie 2016 class. Data is tabulated by: Participant ID, time (pre- vs post-recitation), reported gender, reported first generation college student status, reported if taken BIO340 at ASU, reported ethnicity, recitation time and TA pair, and recitation score.

Sfile5_BIO345_DEMOGRAPHICS_2017_GENIE **Additional file 5.** Text file containing demographic data of participants in the BIO345 Genie 2017 class. Data is tabulated by: Participant ID, time (pre- vs post-recitation), reported gender, reported first generation college student status, reported if taken BIO340 at ASU, reported ethnicity, recitation time and TA pair, and recitation score.

Sfile6_BIO345_DEMOGRAPHICS_2017_NOGENIE **Additional file 6**. Text file containing demographic data of participants in the BIO345 Non-Genie 2017 class. Data is tabulated by: Participant ID, time (pre- vs post-recitation), reported gender, reported first generation college student status, reported if taken BIO340 at ASU, reported ethnicity, recitation time and TA pair, and recitation score.

Sfile7_BIO345_DEMOGRAPHICS_2016 **Additional file 7.** Text file containing demographic data of participants in the BIO345 2016 class. Data is tabulated by: Participant ID, reported gender, reported first generation college student status, reported if taken BIO340 at ASU, reported ethnicity, recitation time and TA pair, pre-recitation score, post-recitation score, and difference between pre- and post-recitation score.

Sfile8_BIO345_DEMOGRAPHICS_2017 **Additional file 8.** Text file containing demographic data of participants in the BIO345 2017 class (combined Genie and Non-Genie classes). Data is tabulated by: Participant ID, reported gender, reported first generation college student status, reported if taken BIO340 at ASU, reported ethnicity, recitation time and TA pair, pre-recitation score, post-recitation score, and difference between pre- and post-recitation score.

Sfile9_COMBINED_TABLE_FOR_DF **Additional file 9.** Text file containing score data for all participants in the study. Data is tabulated by: Year (Genie 2016, Genie 2017, and Non-Genie 2017), recitation time and TA pair, Participant ID, post-recitation score, and difference between pre- and post-recitation score.

Sfile1_Genie_display **Additional file 1.** Genie application display. The main application contains four components: a. grid showing the cellular automata population, b. control panel, c. graph showing number of alleles in the population at any given time, and d. graph showing frequency of different alleles at any given time.

Sfile10_RESULTS_COMPILED_BY_QUESTION **Additional file 10.** Text file containing score data for all participants in the study organized by question. Pre- and post-recitation assessment are listed for each of the 22 question in the Genetic Drift Inventory developed by Price et al. (CBE Life Sci Educ 13(1):65–75, 2014). Correct answers are marked with one (1) and incorrect answers with zero (0). Results are shown for each participant.

Sfile11_VIOLINPLOT_2016_PRE_POST **Additional file 11.** Text file containing the score data used to generate Genie 2016 violin plots. Data is tabulated by year (pre- vs. post-recitation), instruction (Genie vs. Non-Genie), recitation time and TA pair, and participant score.

Sfile12_VIOLINPLOT_2017_GENIE_PRE_POST **Additional file 12.** Text file containing the score data used to generate Genie 2017 violin plots. Data is tabulated by year (pre- vs. post-recitation), instruction (Genie vs. Non-Genie), recitation time and TA pair, and participant score.

Sfile13_VIOLINPLOT_2017_NOGENIE_PRE_POST **Additional file 13.** Text file containing the score data used to generate Non-Genie 2017 violin plots. Data is tabulated by year (pre- vs. post-recitation), instruction (Genie vs. Non-Genie), recitation time and TA pair, and participant score.

Sfile14_violin_plots **Additional file 14.** Figure showing pre- and post-recitation scores changes for individual participants within each class.

Sfile15_results_without_honors_sections **Additional file 15.** Excel file showing the results of the analyses following the removal of honor students. Major results shown as tables and figures in the main text are listed as individual tabs within the file.

Sfile2_BIO345_Spring2016_RECITATION **Additional file 2.** Recitation slides used during BIO345 recitation of Spring 2016. The slides describe basic concepts of genetic drift, introduce Genie features, and provide a list of class activities and driving questions for students to explore.

## Figures and Tables

**Table 1:** Demographic breakdowns of participants in each year and section shows variable representation of different groups

Categories	Genie 2016	Genie 2017	Non-Genie 2017
POC	168	136	120
White	238	144	112
Female	230	190	140
Male	176	90	92
Not first-generation college	280	234	196
First-generation college	126	46	36

The breakdown of participants in each year of the class who participated in the assessment, including those who self-identified as people of color (‘POC’) or ‘white’, ‘female’ or ‘male’, and ‘first-generation’ college students or not. POC was created by combining students identifying as one or more of the following ethnicities: ‘American Indian, Native American, or Alaskan Native’, ‘Asian’, ‘Black or African American’, ‘Hispanic or Latino’, and ‘Native Hawaiian or Other Pacific Islander’

**Table 2:** Most demographic predictors did not affect performance on the evaluation of genetic drift knowledge

Query	Predictor	Df	F-value	Corrected p-values
Pre-recitation scores dependence on demographic variables in 2016	First generation college	1	4.142	0.303
	Ethnicity	6	1.046	1
	Gender	1	1.004	1
	First generation college: Ethnicity	5	2.613	0.183
	First generation college: Gender	1	0.242	1
	Ethnicity: Gender	4	1.373	1
	First generation college: Ethnicity: Gender	3	0.912	1
	Residuals	181		
Post-recitation scores dependence on demographic variables in 2016	First generation college	1	7.955	0.037^±^
	Ethnicity	6	0.372	1
	Gender	1	2.388	0.868
	First generation college: Ethnicity	5	2.949	0.097
	First generation college: Gender	1	0.800	1
	Ethnicity: Gender	4	1.156	1
	First generation college: Ethnicity: Gender	3	0.616	1
	Residuals	181		
Pre-recitation scores dependent on demographic variables in 2017	First generation college	1	0.466	1
	Ethnicity	7	2.286	0.375
	Gender	1	0.037	1
	Genie used	1	6.131	0.183
	First generation college: Ethnicity	5	0.563	1
	First generation college: Gender	1	0.397	1
	Ethnicity: Gender	6	2.120	0.678
	First generation college: Genie used	1	4.096	0.575
	Ethnicity: Genie used	5	1.053	1
	Gender: Genie used	1	9.495	0.030^±^
	First generation college: Ethnicity: Gender	3	1.653	1
	First generation college: Ethnicity: Genie used	3	1.846	1
	Ethnicity: Gender: Genie used	3	1.155	1
	Residuals	217		
Post-recitation scores dependent on demographic variables in 2017	First generation college	1	0.26	1
	Ethnicity	7	0.895	1
	Gender	1	0.010	1
	Genie used	1	2.350	1
	First generation college: Ethnicity	5	0.409	1
	First generation college: Gender	1	0.485	1
	Ethnicity: Gender	6	1.346	1
	First generation college: Genie used	1	0.757	1
	Ethnicity: Genie used	5	2.062	0.927
	Gender: Genie used	1	1.580	1
	First generation college: Ethnicity: Gender	3	0.486	1
	First generation college: Ethnicity: Genie used	3	1.902	1
	Ethnicity: Gender: Genie used	3	0.454	1
	Residuals	217		

The general linear regression of pre- and post-recitation scores with demographic predictors, the degrees of freedom (Df), and summary statistics. All p-values have been corrected using a Bonferroni

± Significant p-values

**Table 3:** Post-recitation scores were mostly influenced by pre-recitation scores, but not class section

Query	Predictor	SE	z-score	p-value
Pre-recitation scores differences across Genie 2016, Genie 2017, and Non-Genie 2017	Genie 2017	0.104	9.241	< 2 × 10^−16^^±^
	Non-Genie 2017	0.102	5.081	3.75 × 10^−07^^±^
Post-recitation scores dependence on the pre-recitation scores and class (Genie 2016, Genie 2017, and Non-Genie 2017)	Pre-recitation scores	0.237	14.55	< 2 × 10^−16^^±^
	Genie 2017	0.103	4.948	7.51 × 10^−^^07^^±^
	Non-Genie 2017	0.099	2.847	0.004^±^
Post-recitation scores of 2016 dependence on the pre-recitation scores and the class section	Pre-recitation score	0.391	8.17	3.08 × 10^−16^^±^
	TA Pair1 4:30 pm	0.217	−1.764	0.078
	TA Pair1 6:00 pm	0.233	−0.702	0.483
	TA Pair1 7:30 pm	0.285	−2.387	0.017^±^
	TA Pair2 3:00 pm	0.226	1.366	0.172
	TA Pair2 4:30 pm	0.205	−0.468	0.64
	TA Pair2 6:00 pm	0.234	−1.433	0.152
	TA Pair2 7:30 pm	0.242	0.213	0.831
Post-recitation scores dependence on pre-recitation scores and Genie 2017class sections	Pre-recitation score	0.452	7.797	6.64 × 10^−15^^±^
	TA Pair1 7:00 pm	0.21	−0.996	0.319
	TA Pair2 1:30 pm	0.209	−0.152	0.879
	TA Pair2 7:00 pm	0.223	0.026	0.98
Post-recitation scores dependence on pre-recitation scores and Non-Genie 2017 class sections	Pre-recitation score	0.37	9.695	< 2 × 10^−^^16^^±^
	TA Pair1 4:30 pm	0.194	−0.116	0.907
	TA Pair2 3:00 pm	0.188	1.75	0.08
	TA Pair2 4:30 pm	0.19	−0.659	0.509

The Beta regression tests of pre- and post-recitation scores for specific queries, including the predictors, standard errors (SE), z-scores, and p-values

± Significant p-values

**Table 4:** Effect of Genie on learning outcomes

Query	Cohen’s d (Lower 95% CI, Upper 95% CI)
Pre- vs post-recitation scores in Genie 2016	0.608 (0.408, 0.807)
Pre- vs post-recitation scores in Genie 2017	0.632 (0.410, 0.855)
Pre- vs post-recitation scores in Non-Genie 2017	0.658 (0.430, 0.886)
Pre-recitation scores in Genie 2017 vs Non-Genie 2017	0.272 (0.051, 0.493)
Post-recitation scores in Genie 2017 vs Non-Genie 2017	0.242 (0.021, 0.462)
Difference in pre- and post-recitation scores between Genie 2017 and Non-Genie 2017	− 0.087 (− 0.307, 0.133)

The size-effect analysis of recitation scores per year

**Table 5:** Individual participants in all classes showed higher post-recitation scores compared to their pre-recitation scores

Query	t -value (Bonferroni p-value)
Paired post- vs pre-recitation scores in Genie 2016	−9.747 (2.2 × 10^−16^)^±^
Paired post- and pre-recitation scores in Genie 2017	−8.966 (6.913 × 10^−16^)^±^
Paired post- and pre-recitation scores in Non-Genie 2017	−9.816 (2.2 × 10^−16^)^±^

± Significant p-values

Paired Student’s t-test for individual participants in pre- and post-recitation scores per class

**Table 6:** For most individual questions, participant post-recitation performance improved across classes following instruction either with or without Genie

Evaluation		Question	Genie 2016		Genie 2017		Non-genie 2017	
			McNemar stat	Bonferroni	McNemar stat	Bonferroni	McNemar stat	Bonferroni
Key concepts		Q1	1.778	1	1	1	3.556	1
		Q3	35.267	6.34 × 10^−08±^	6.25	0.264	2.667	1
		Q15	2.951	1	3.24	1	7.348	0.154
		Q4	16.86	8.84 × 10^−04^^±^	0.862	1	0.36	1
		Q10	2.174	1	9.143	0.044^±^	8.909	0.066^±^
		Q13	62.411	6.14 × 10^−14±^	19.703	1.99 × 10^−04±^	30.857	6.12 × 10^−07±^
		Q16	3.169	1	3.13	1	0.158	1
Misconceptions*	1	Q7	3.314	1	4.235	0.880	5.121	0.528
	2	Q5	19.636	2.06 × 10^−04^^±^	16.892	8.71 × 10^−04±^	28.488	2.07 × 10^−06±^
		Q6	16.056	1.35 × 10^−03^^±^	33.923	1.26 × 10^−07±^	46.538	1.98 × 10^−10±^
		Q8	8.471	0.088	16.953	8.43 × 10^−04±^	24.923	1.313 × 10^−05±^
	3	Q2	2.882	1	3.457	1	0	1
	4	Q9	1	1	8.167	0.088	7.538	0.132
		Q12	17.61	5.96 × 10^−04^^±^	16.892	8.71 × 10^−04±^	11.756	0.013^±^
		Q17	30.229	8.45 × 10^−07^^±^	26.843	4.86 × 10^−06±^	22.73	4.09 × 10^−05±^
		Q20	8.345	0.088	13.37	5.63 × 10^−03±^	6.368	0.264
	5	Q14	2.513	1	12.737	7.90 × 10^−03±^	9.091	0.066
		Q19	0.12	1	0.034	1	6.081	0.308
		Q22	0	1	6.125	0.286	9.966	0.044
	6	Q11	16.254	0.001^±^	8.533	0.066	19.703	1.99 × 10^−04±^
		Q18	11.919	0.012^±^	5.556	0.396	4.545	0.726
		Q21	0.308	1	8.067	1	1.2	1

McNemar’s test performed on (in)correct to (in)correct pre- and post-recitation answers per question. McNemar statistics and Bonferroni corrected p-values are provided. We show this for Genie 2016, Genie 2017, and Non-Genie 2017

± Significant p-values

* Misconceptions (Price et al. 2014)

1. Genetic drift is unpredictable because it has a random component

2. Genetic drift is natural selection/adaptation/acclimation to the environment that may result from a need to survive

3. Genetic drift is not evolution because it does not lead to directional change that increases fitness

4. Natural selection is always the most powerful mechanism of evolution, and it is the primary agent of evolutionary change

5. Genetic drift is random mutation

6. Genetic drift is gene flow or migration

**Table 7:** Comparison of performance between Genie 2017 and Non-Genie 2017, controlled by question

Evaluation		Question	Genie 2017		Non-Genie 2017		OR.est	p-value
			Incorrect to correct switches	Correct to incorrect switches	Incorrect to correct switches	Correct to incorrect switches		
Key concepts		Q1	6	3	13	5	0.777	1.000
		Q3	13	3	16	8	2.126	0.473
		Q15	17	8	18	5	0.597	0.523
		Q4	17	12	14	11	1.111	1.000
		Q10	22	6	18	4	0.818	1.000
		Q13	32	5	39	3	0.497	0.463
		Q16	29	17	30	27	1.529	0.321
Misconceptions*	1	Q7	23	11	23	10	0.910	1.000
	2	Q5	31	6	39	4	0.534	0.501
		Q6	47	5	60	5	0.785	0.749
		Q8	35	8	44	8	0.797	0.785
	3	Q2	23	12	20	20	1.900	0.242
	4	Q9	19	5	20	6	1.137	1.000
		Q12	31	6	34	11	1.661	0.421
		Q17	44	7	33	4	0.764	0.755
		Q20	23	4	15	4	1.519	0.700
	5	Q14	30	8	32	12	1.400	0.610
		Q19	15	14	26	11	0.459	0.136
		Q22	23	9	23	6	0.671	0.562
	6	Q11	23	7	32	5	0.519	0.349
		Q18	14	4	16	6	1.304	1.000
		Q21	13	2	18	12	4.207	0.094

Fisher’s exact test testing the association between switches from ‘Incorrect to Correct’ and ‘Correct to Incorrect’ answers per question and by method of instruction (Genie 2017 and Non-Genie 2017)

± Significant p-values

* Misconceptions (Price et al. 2014)

1. Genetic drift is unpredictable because it has a random component

2. Genetic drift is natural selection/adaptation/acclimation to the environment that may result from a need to survive

3. Genetic drift is not evolution because it does not lead to directional change that increases fitness

4. Natural selection is always the most powerful mechanism of evolution, and it is the primary agent of evolutionary change

5. Genetic drift is random mutation

6. Genetic drift is gene flow or migration

## Data Availability

IRB protocol: STUDY00003707. A previous version of this manuscript is available as preprint (https://doi.org/10.1101/268672). All data and code used has been made available as additional files. Genie is publicly available at https://cartwrig.ht/apps/genie/.
