# Supplementary figures and images for: Genie: an interactive real-time simulation for teaching genetic drift
Source: Evolution (N Y). Author manuscript; Available in PMC 2022 Oct 12. (PMC9555832; doi:10.1186/s12052-022-00161-7)

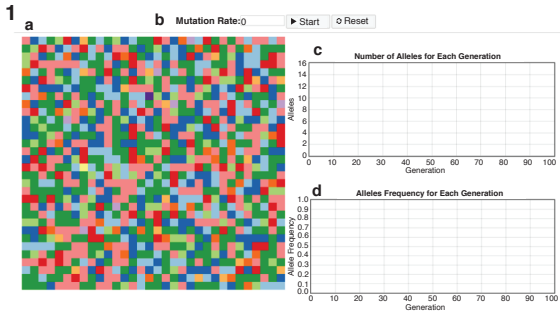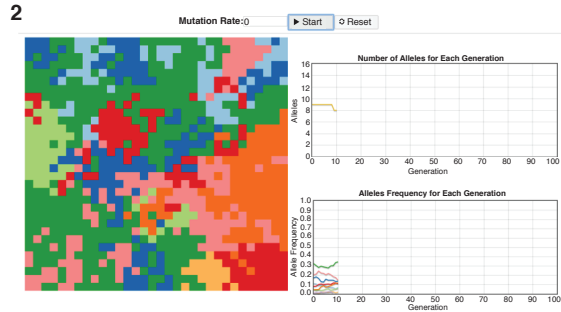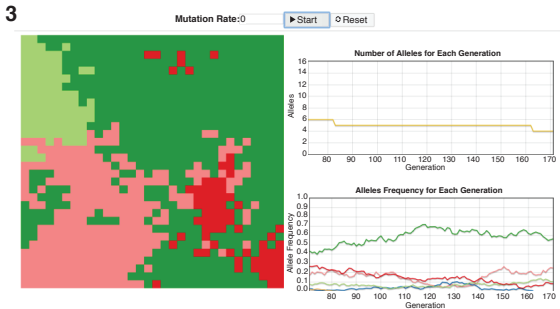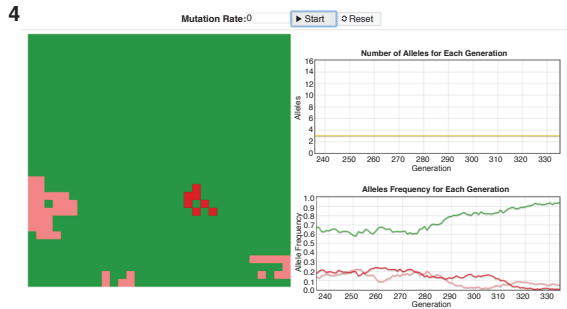

Supplement: Sfile1_Genie_display — Additional file 1. Genie application display. The main application contains four components: a. grid showing the cellular automata population, b. control panel, c. graph showing number of alleles in the population at any given time, and d. graph showing frequency of different alleles at any given time. [file NIHMS1805352-supplement-Sfile1_Genie_display.pdf]

Genie 2016

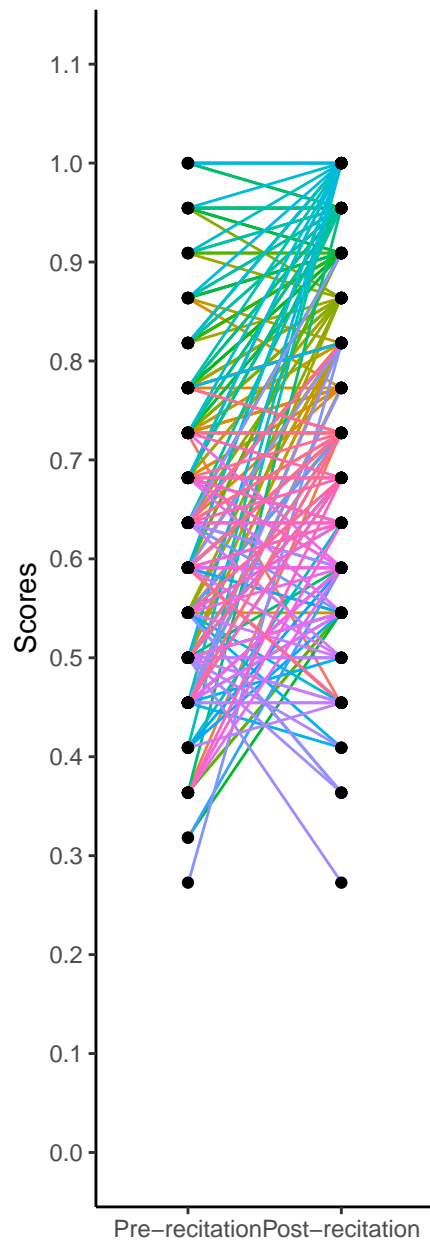

Genie 2017

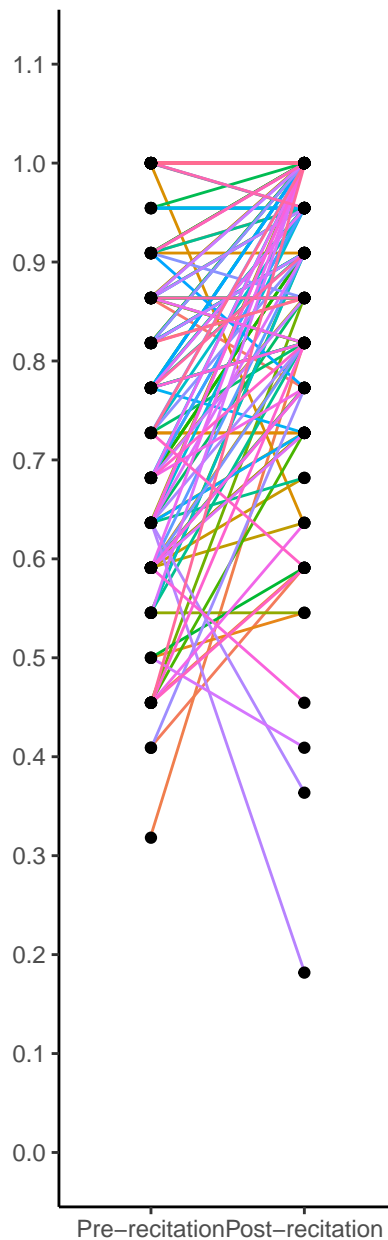

Non-Genie 2017

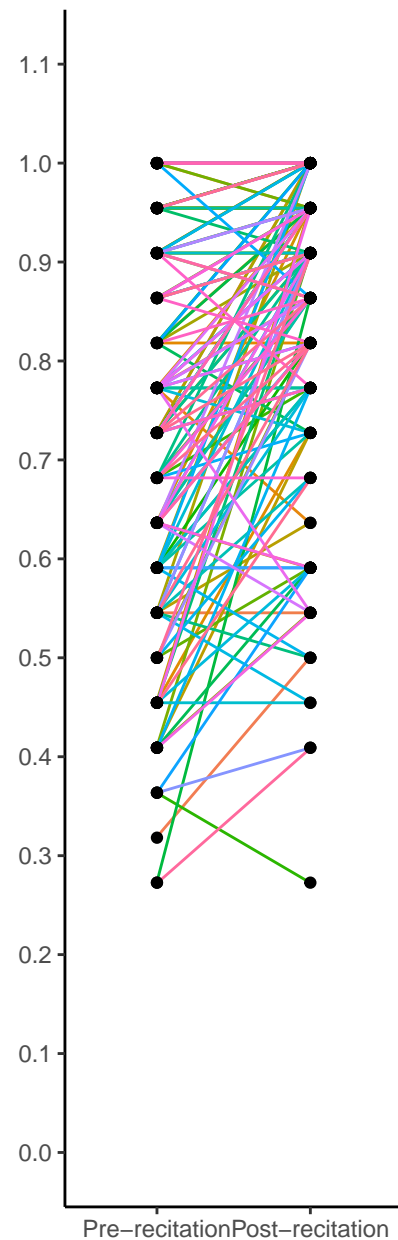

Supplement: Sfile14_violin_plots — Additional file 14. Figure showing pre- and post-recitation scores changes for individual participants within each class. [file NIHMS1805352-supplement-Sfile14_violin_plots.pdf]
