# Supplementary material for: Genie: an interactive real-time simulation for teaching genetic drift
Source: Evolution (N Y). Author manuscript; Available in PMC 2022 Oct 12. (PMC9555832; doi:10.1186/s12052-022-00161-7)
Supplement: Sfile2_BIO345_Spring2016_RECITATION — Additional file 2. Recitation slides used during BIO345 recitation of Spring 2016. The slides describe basic concepts of genetic drift, introduce Genie features, and provide a list of class activities and driving questions for students to explore. [file NIHMS1805352-supplement-Sfile2_BIO345_Spring2016_RECITATION.pptx]

## Slide 1
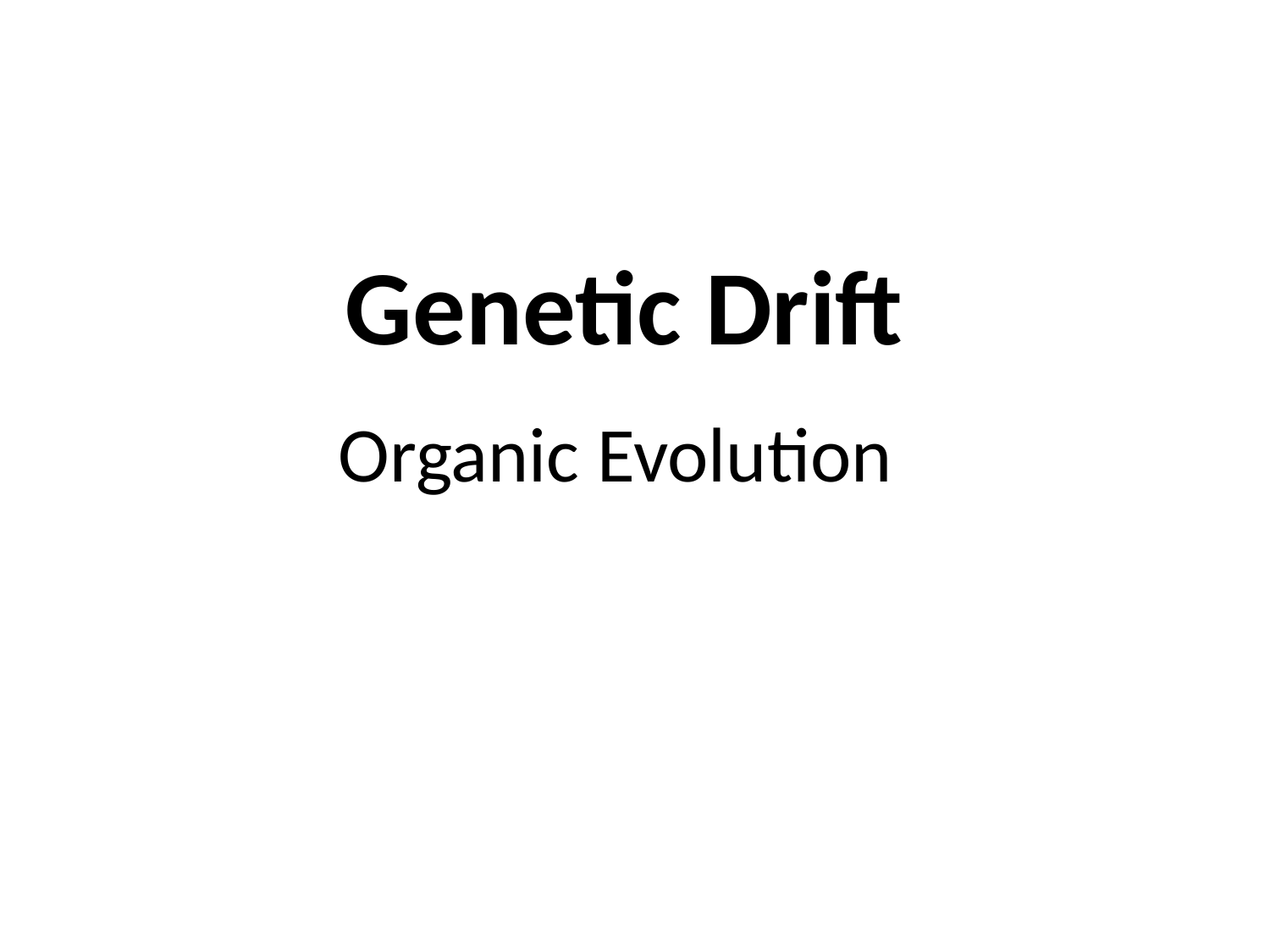

Genetic Drift
Organic Evolution

## Slide 2
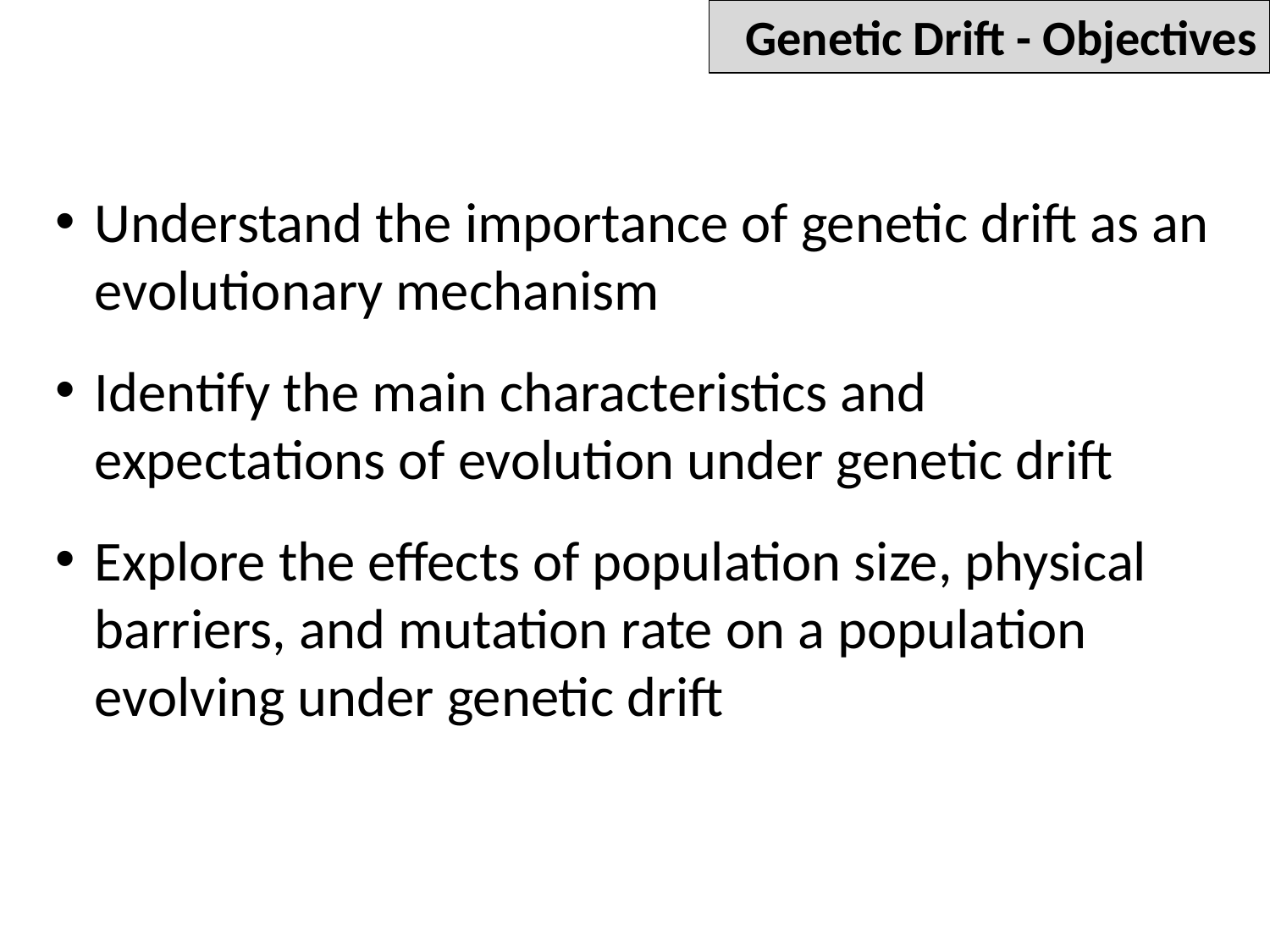

Genetic Drift - Objectives
Understand the importance of genetic drift as an evolutionary mechanism
Identify the main characteristics and expectations of evolution under genetic drift
Explore the effects of population size, physical barriers, and mutation rate on a population evolving under genetic drift

## Slide 3
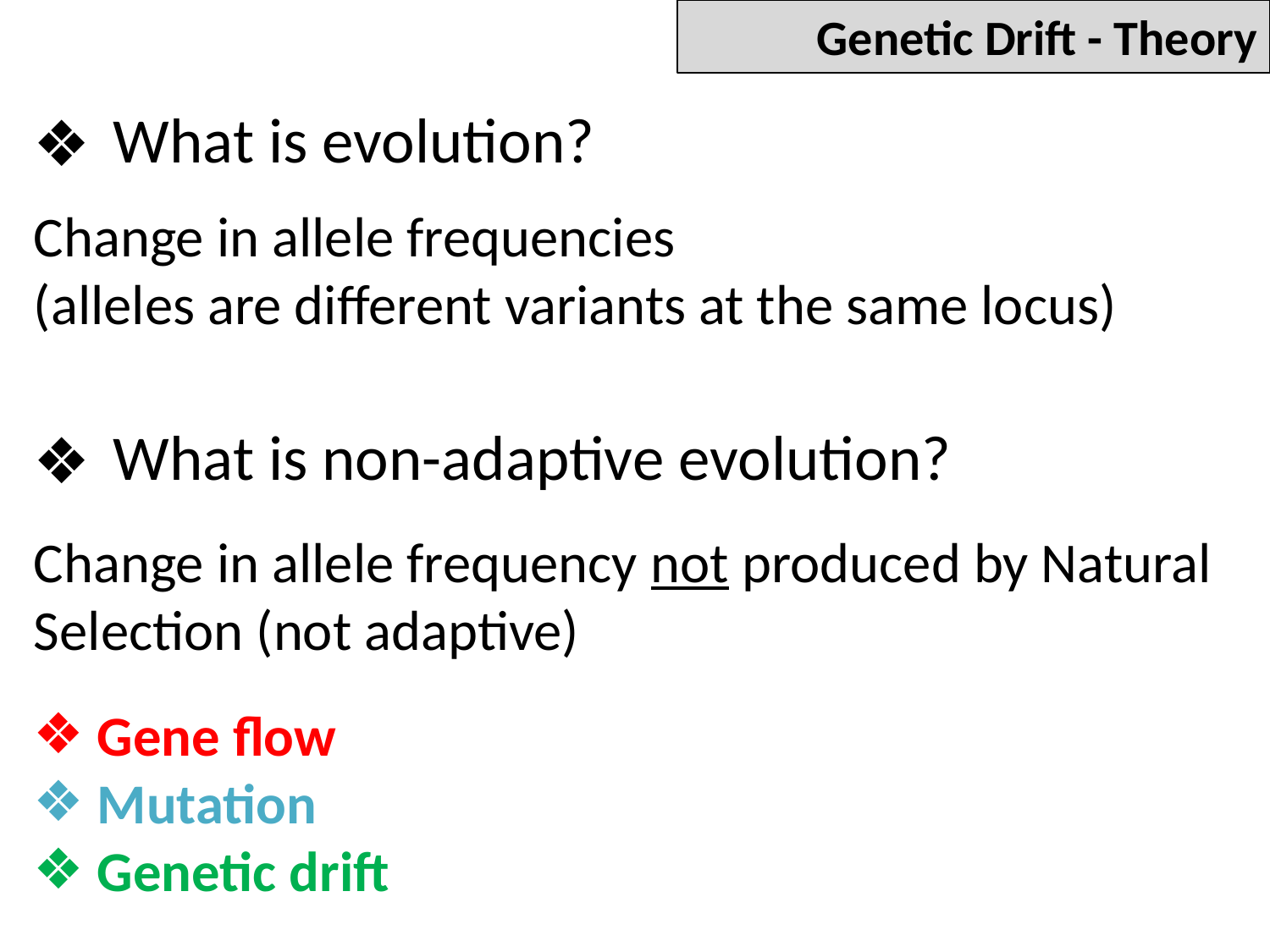

Genetic Drift - Theory
What is evolution?
Change in allele frequencies
(alleles are different variants at the same locus)
What is non-adaptive evolution?
Change in allele frequency not produced by Natural Selection (not adaptive)
Gene flow
Mutation
Genetic drift

## Slide 4
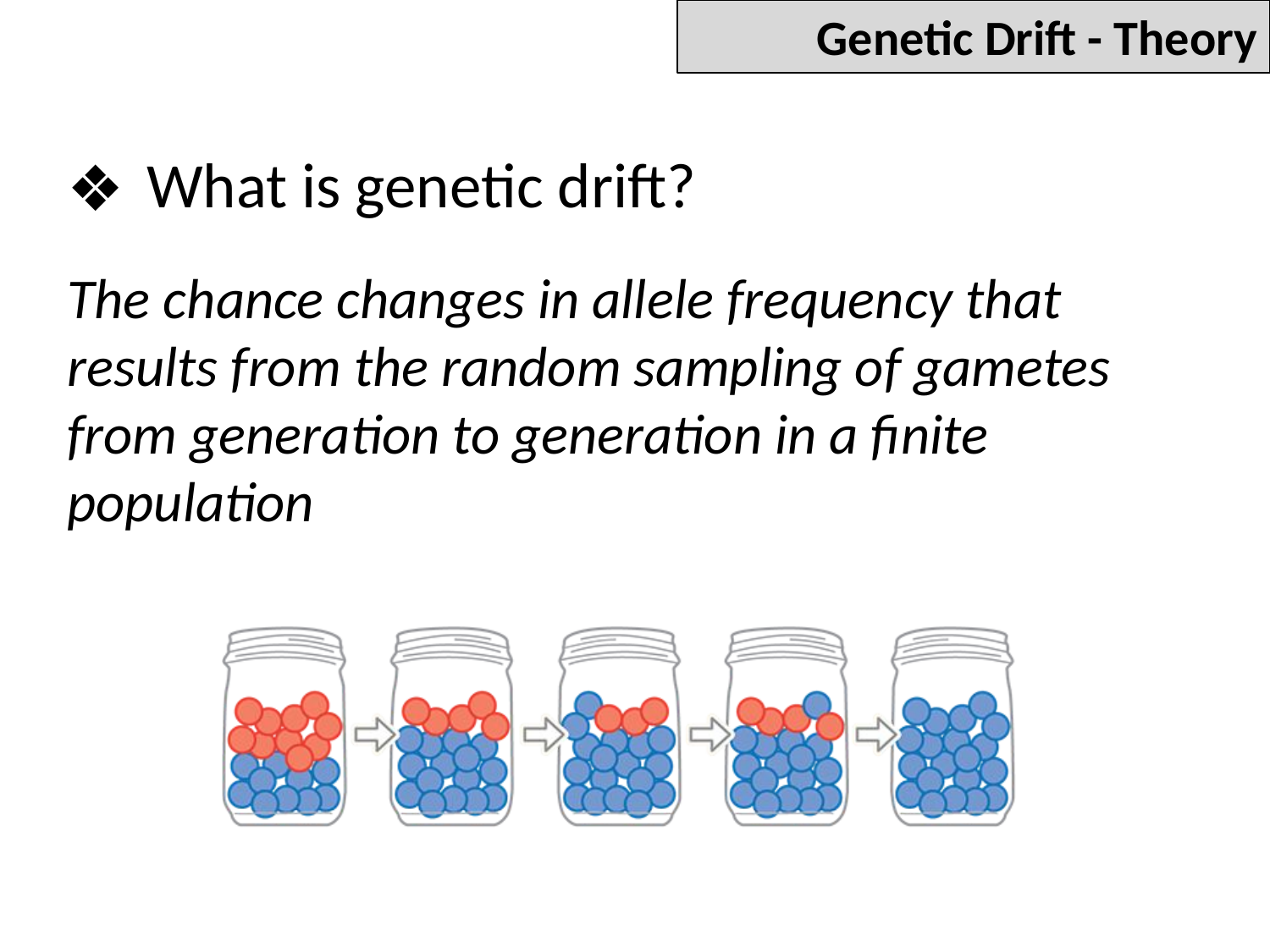

Genetic Drift - Theory
What is genetic drift?
The chance changes in allele frequency that results from the random sampling of gametes from generation to generation in a finite population

## Slide 5
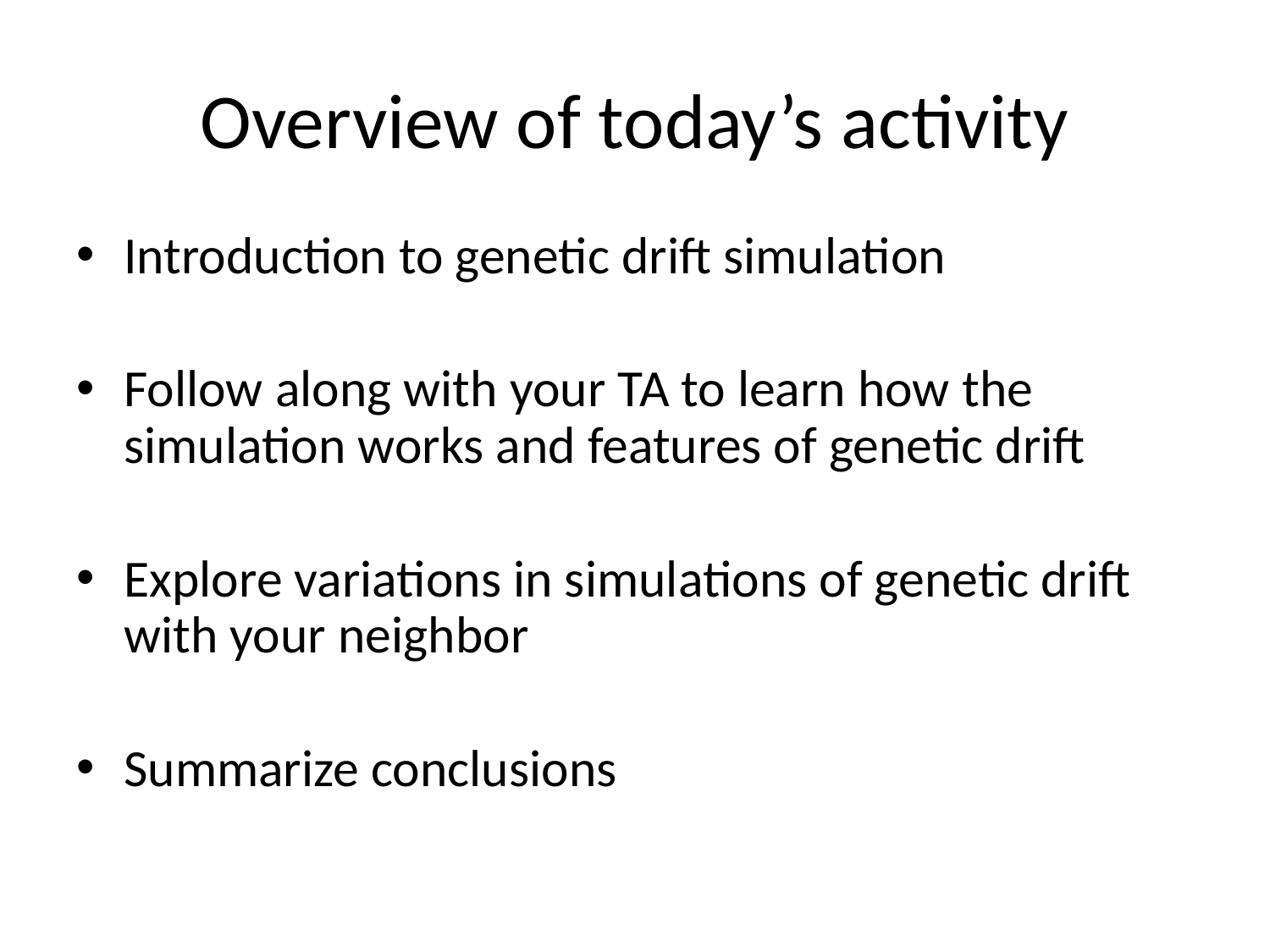

# Overview of today’s activity
Introduction to genetic drift simulation
Follow along with your TA to learn how the simulation works and features of genetic drift
Explore variations in simulations of genetic drift with your neighbor
Summarize conclusions

## Slide 6
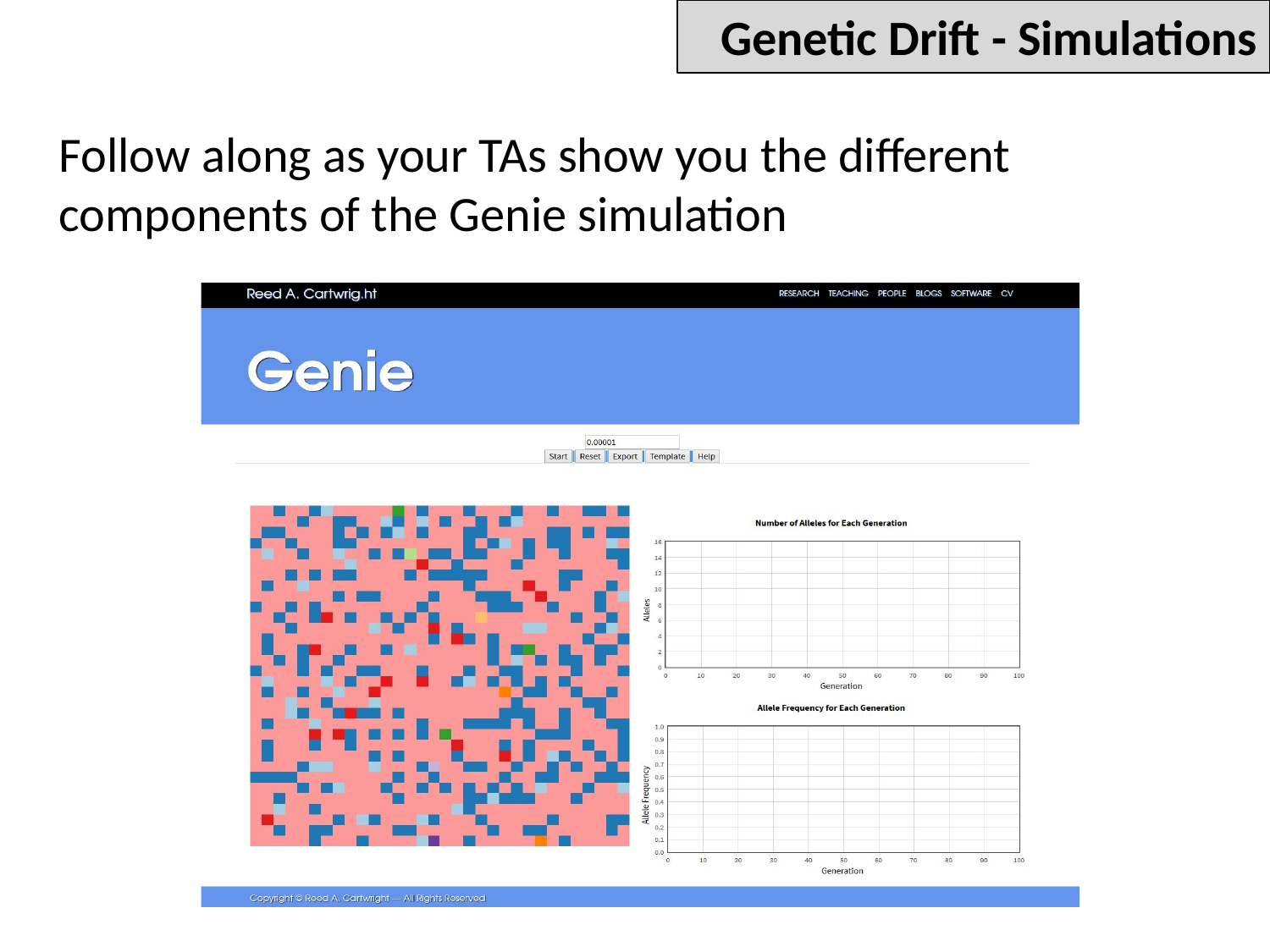

Genetic Drift - Simulations
Follow along as your TAs show you the different components of the Genie simulation

## Slide 7
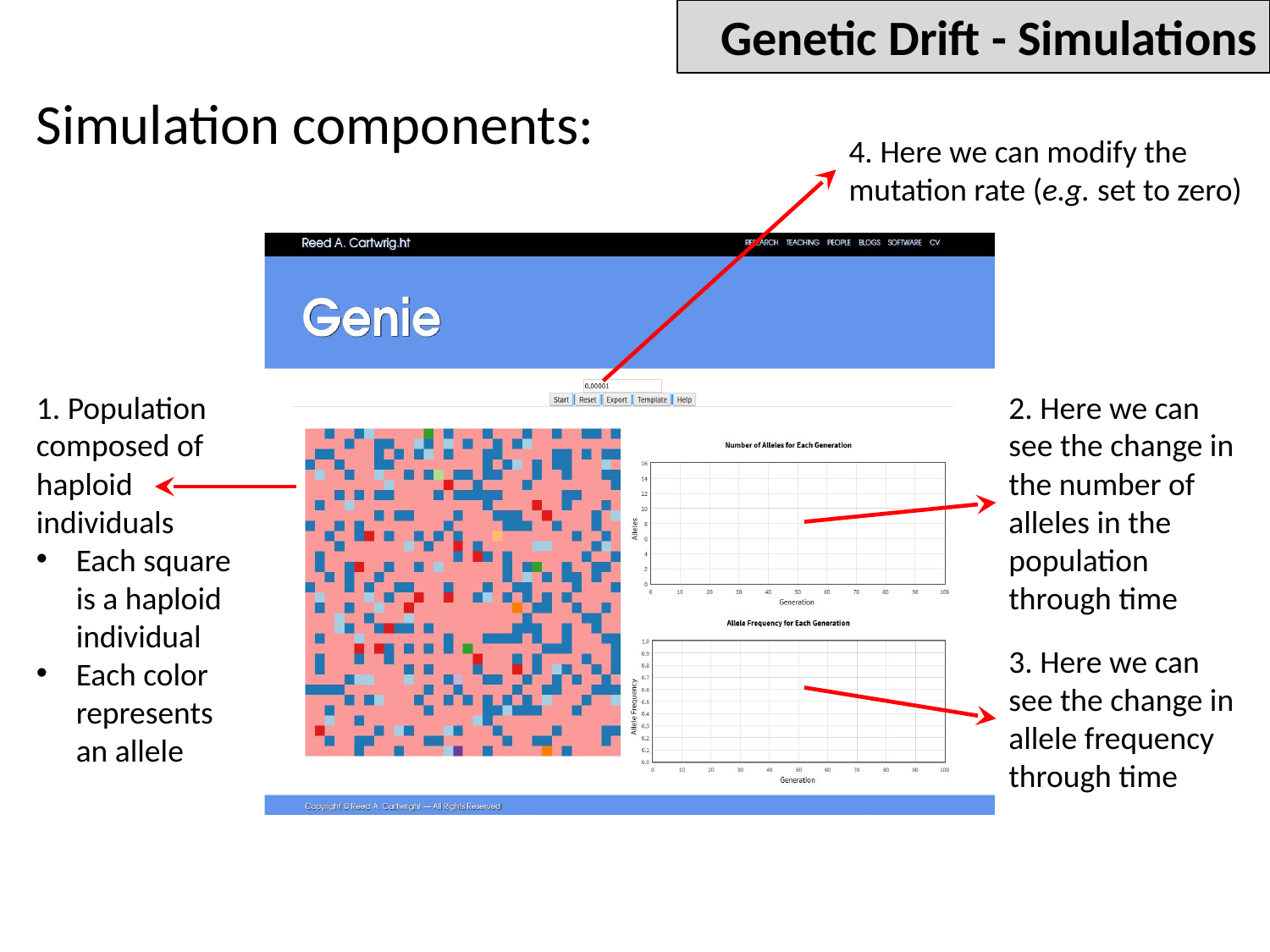

Genetic Drift - Simulations
Simulation components:
4. Here we can modify the mutation rate (e.g. set to zero)
1. Population composed of haploid individuals
Each square is a haploid individual
Each color represents an allele
2. Here we can see the change in the number of alleles in the population through time
3. Here we can see the change in allele frequency through time

## Slide 8
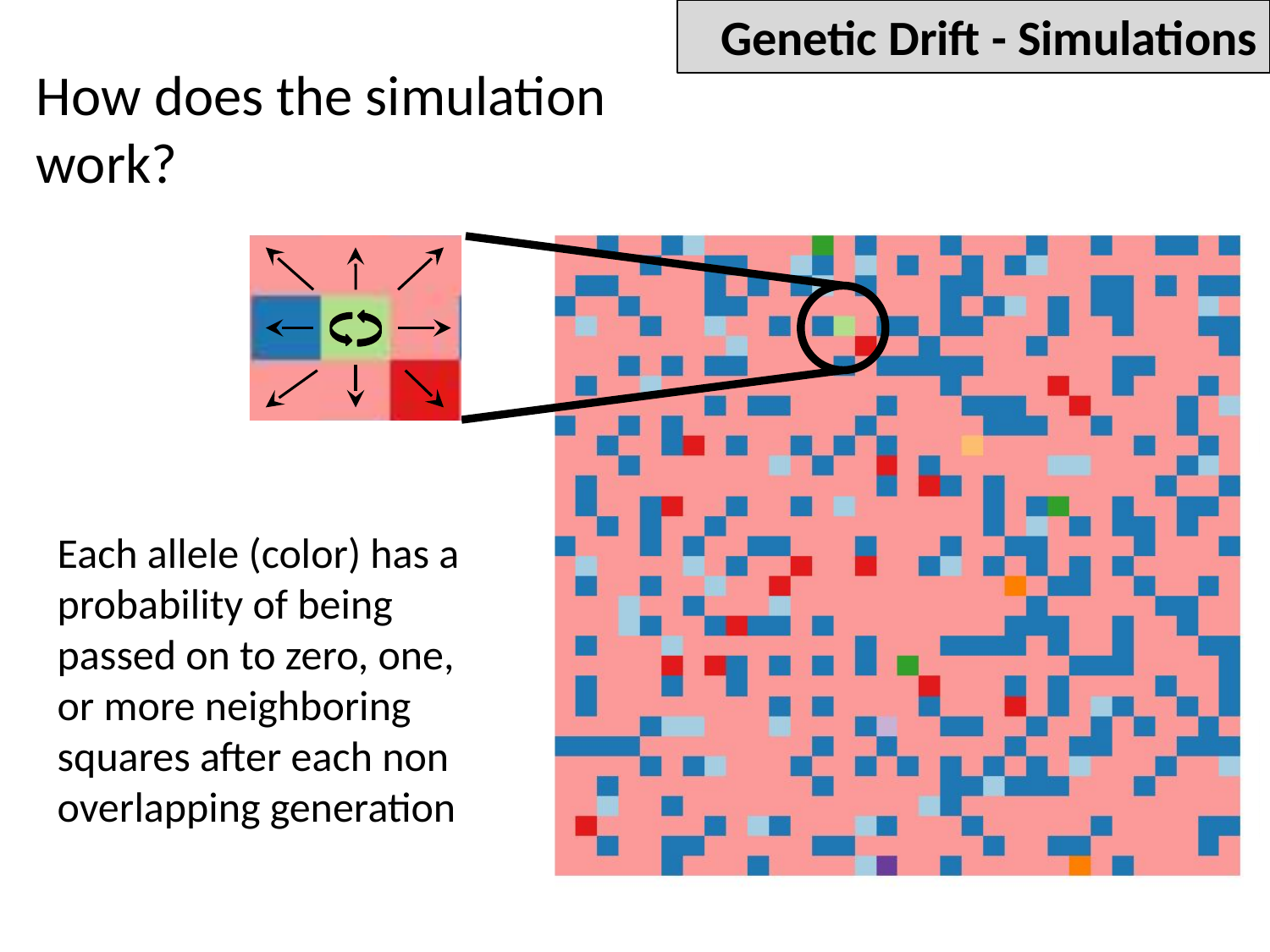

Genetic Drift - Simulations
How does the simulation work?
Each allele (color) has a probability of being passed on to zero, one, or more neighboring squares after each non­ overlapping generation

## Slide 9
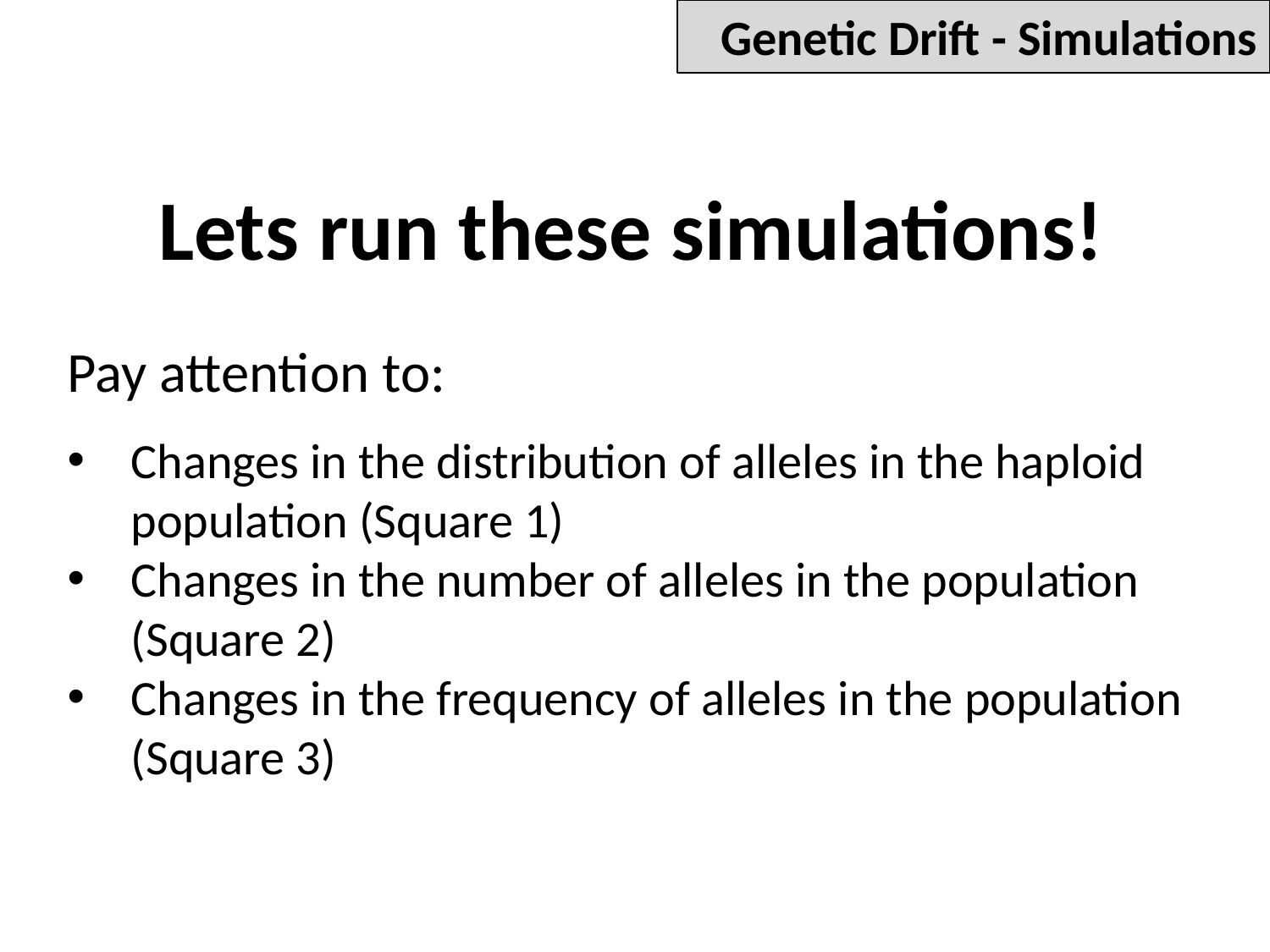

Genetic Drift - Simulations
Lets run these simulations!
Pay attention to:
Changes in the distribution of alleles in the haploid population (Square 1)
Changes in the number of alleles in the population (Square 2)
Changes in the frequency of alleles in the population (Square 3)

## Slide 10
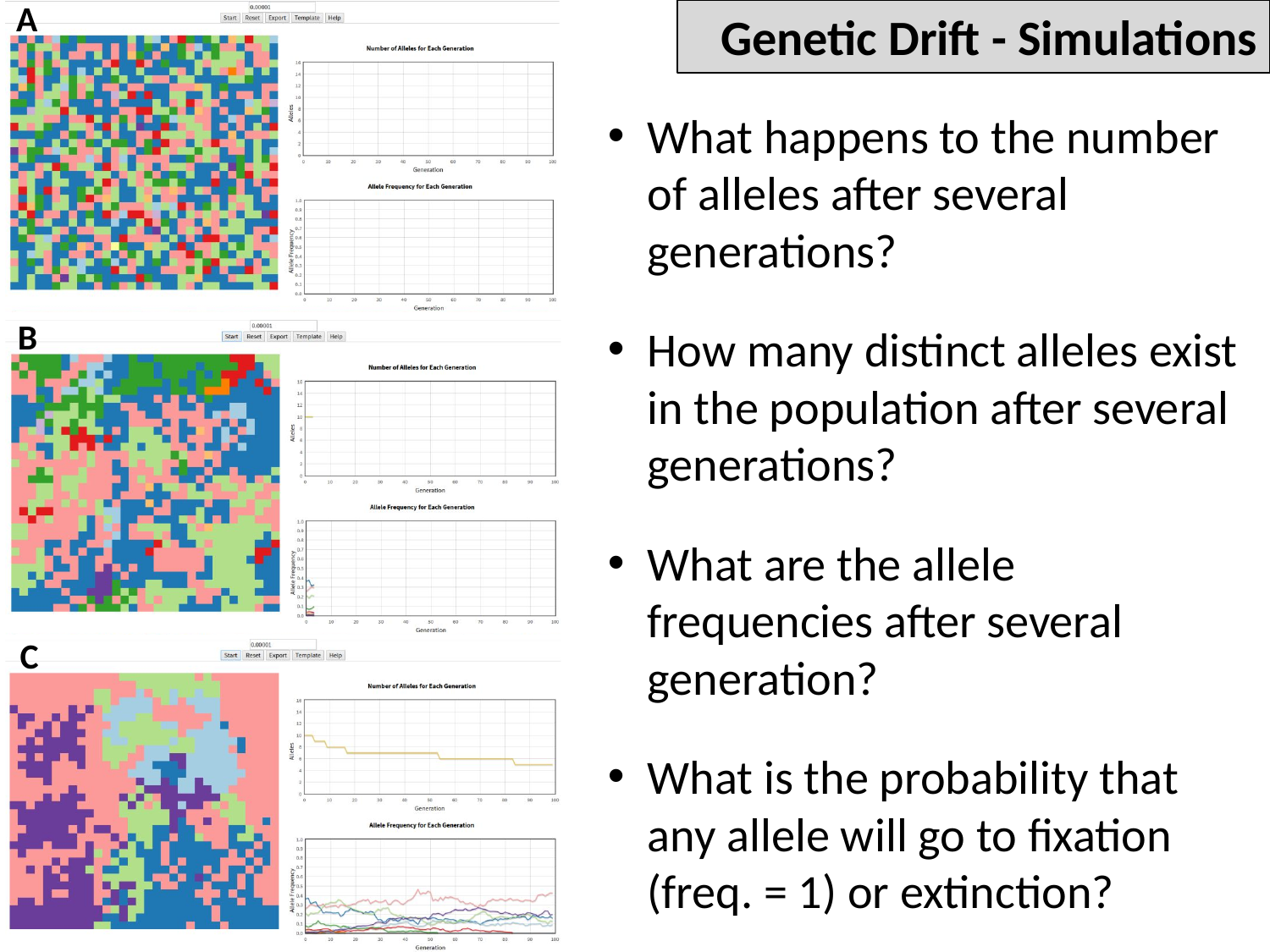

Genetic Drift - Simulations
A
What happens to the number of alleles after several generations?
How many distinct alleles exist in the population after several generations?
What are the allele frequencies after several generation?
What is the probability that any allele will go to fixation (freq. = 1) or extinction?
B
C

## Slide 11
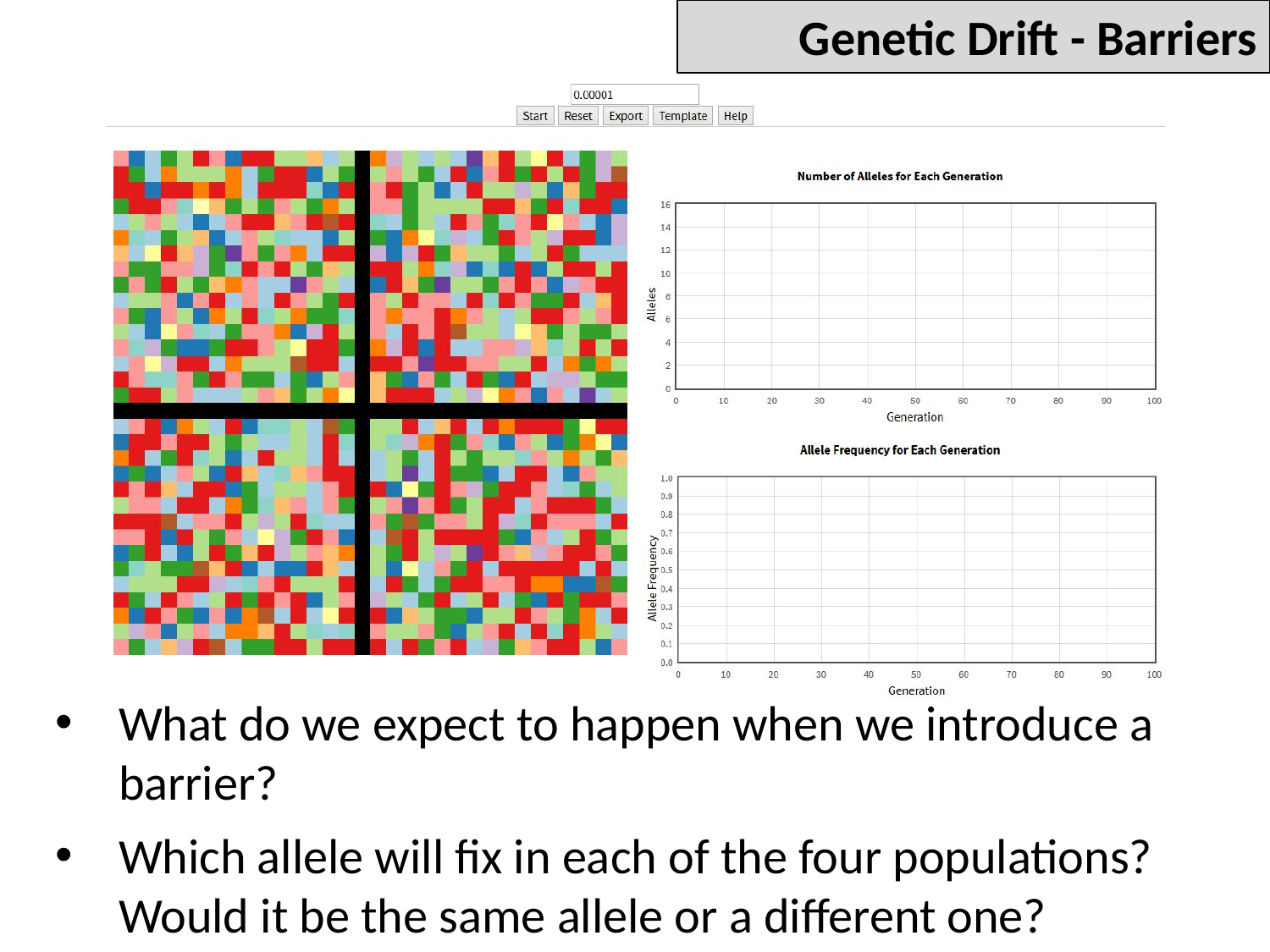

Genetic Drift - Barriers
What do we expect to happen when we introduce a barrier?
Which allele will fix in each of the four populations? Would it be the same allele or a different one?

## Slide 12
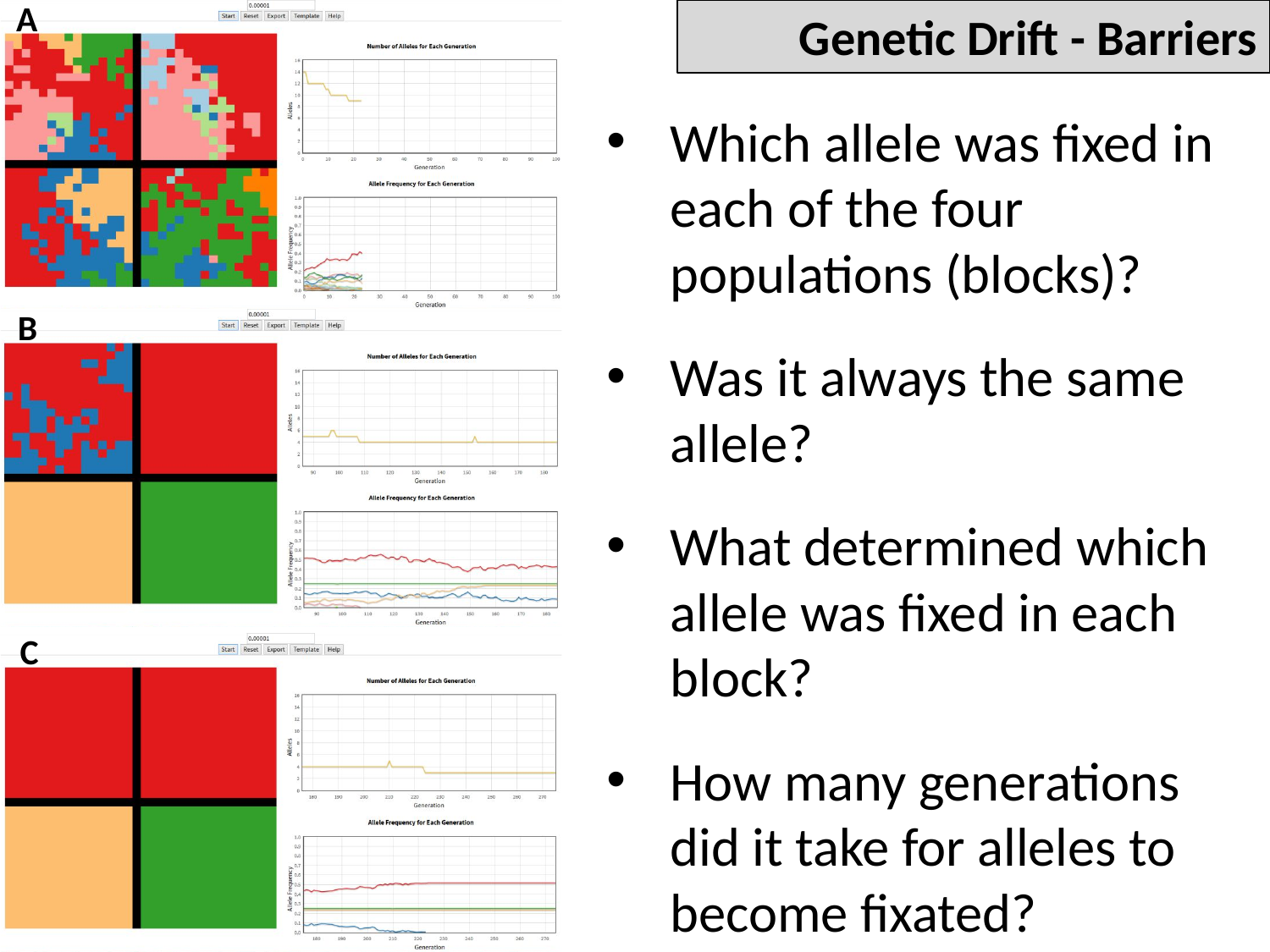

Genetic Drift - Barriers
A
Which allele was fixed in each of the four populations (blocks)?
Was it always the same allele?
What determined which allele was fixed in each block?
How many generations did it take for alleles to become fixated?
B
C

## Slide 13
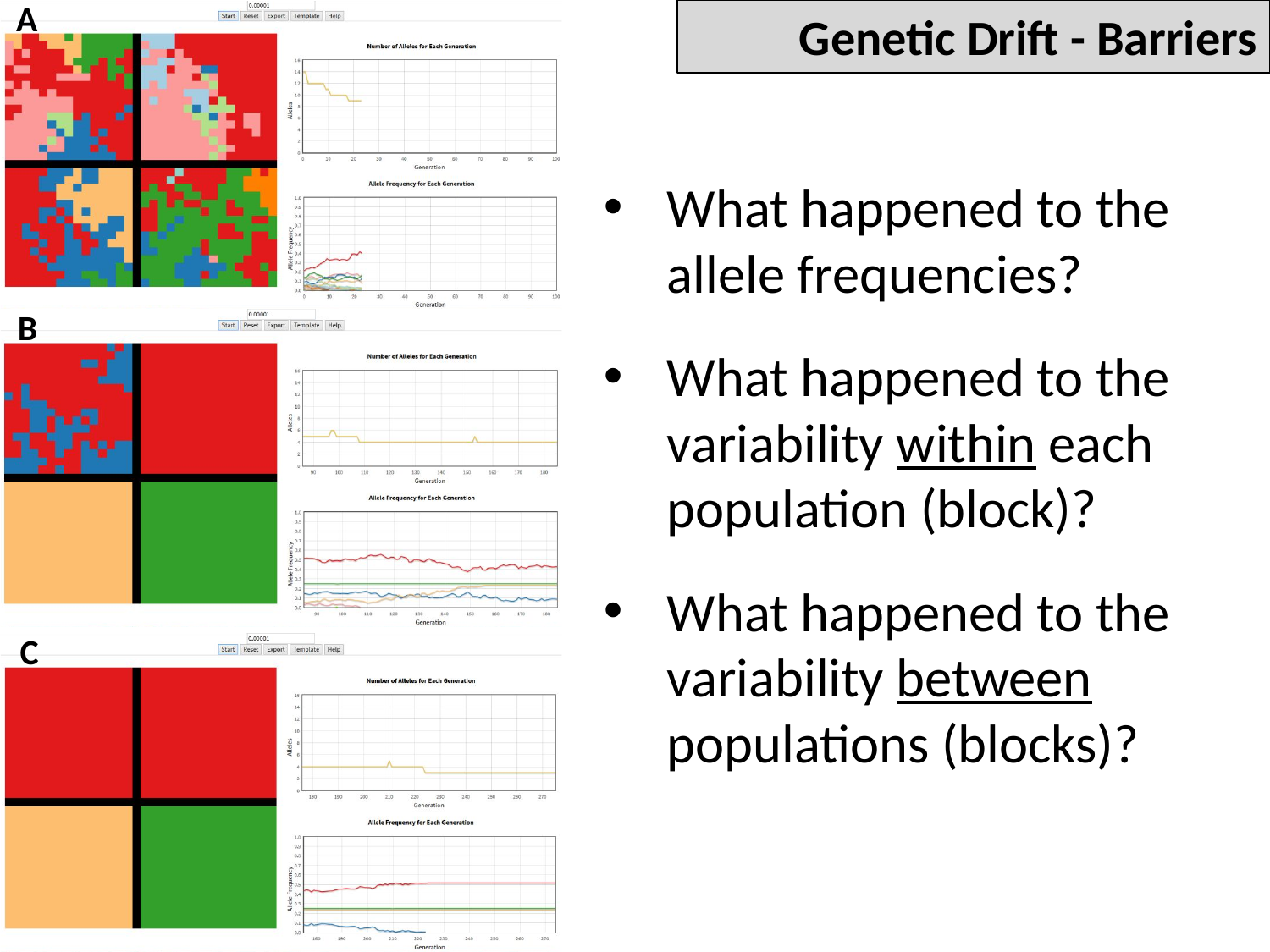

Genetic Drift - Barriers
A
What happened to the allele frequencies?
What happened to the variability within each population (block)?
What happened to the variability between populations (blocks)?
B
C

## Slide 14
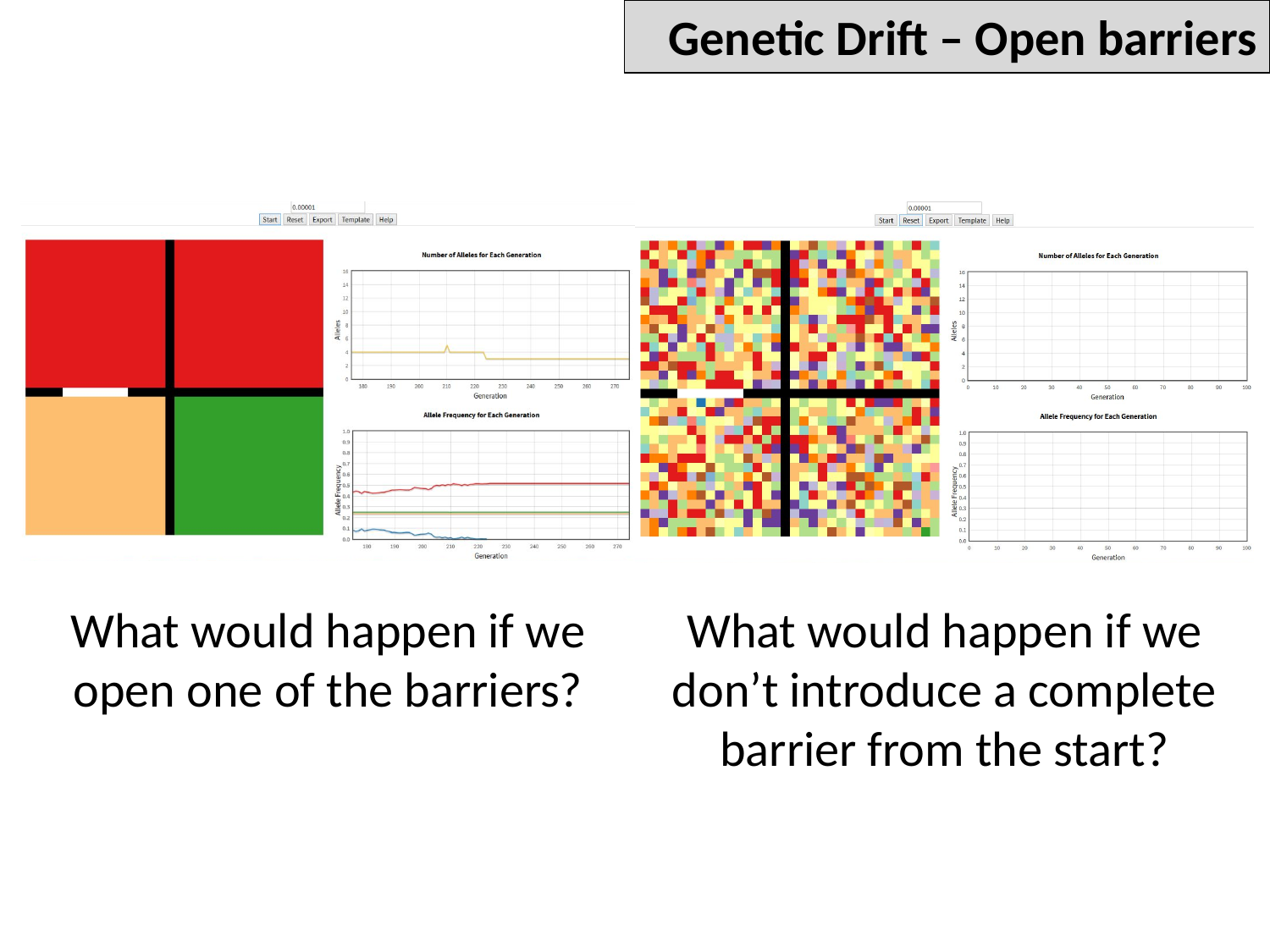

Genetic Drift – Open barriers
What would happen if we don’t introduce a complete barrier from the start?
What would happen if we open one of the barriers?

## Slide 15
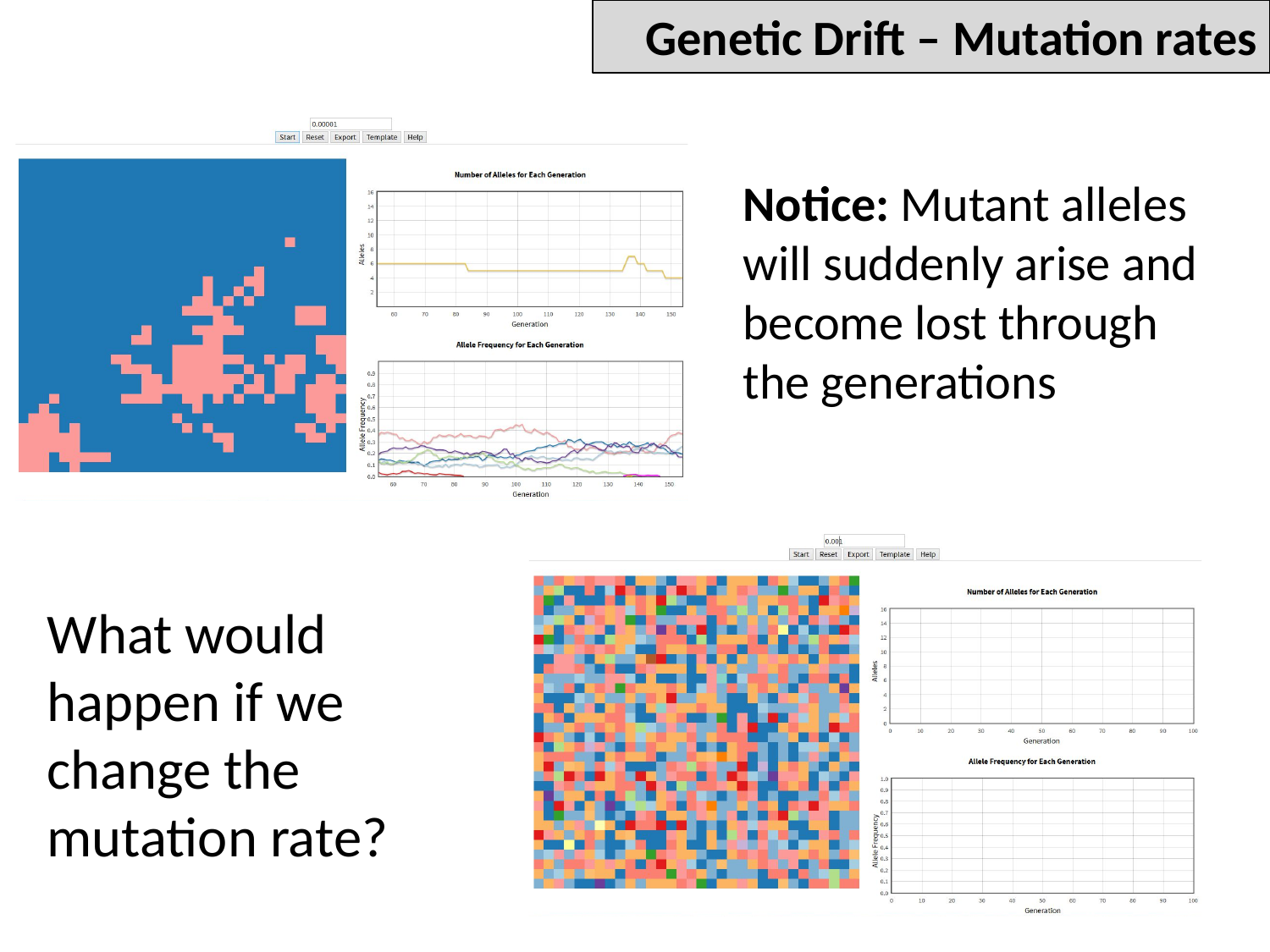

Genetic Drift – Mutation rates
Notice: Mutant alleles will suddenly arise and become lost through the generations
What would happen if we change the mutation rate?

## Slide 16
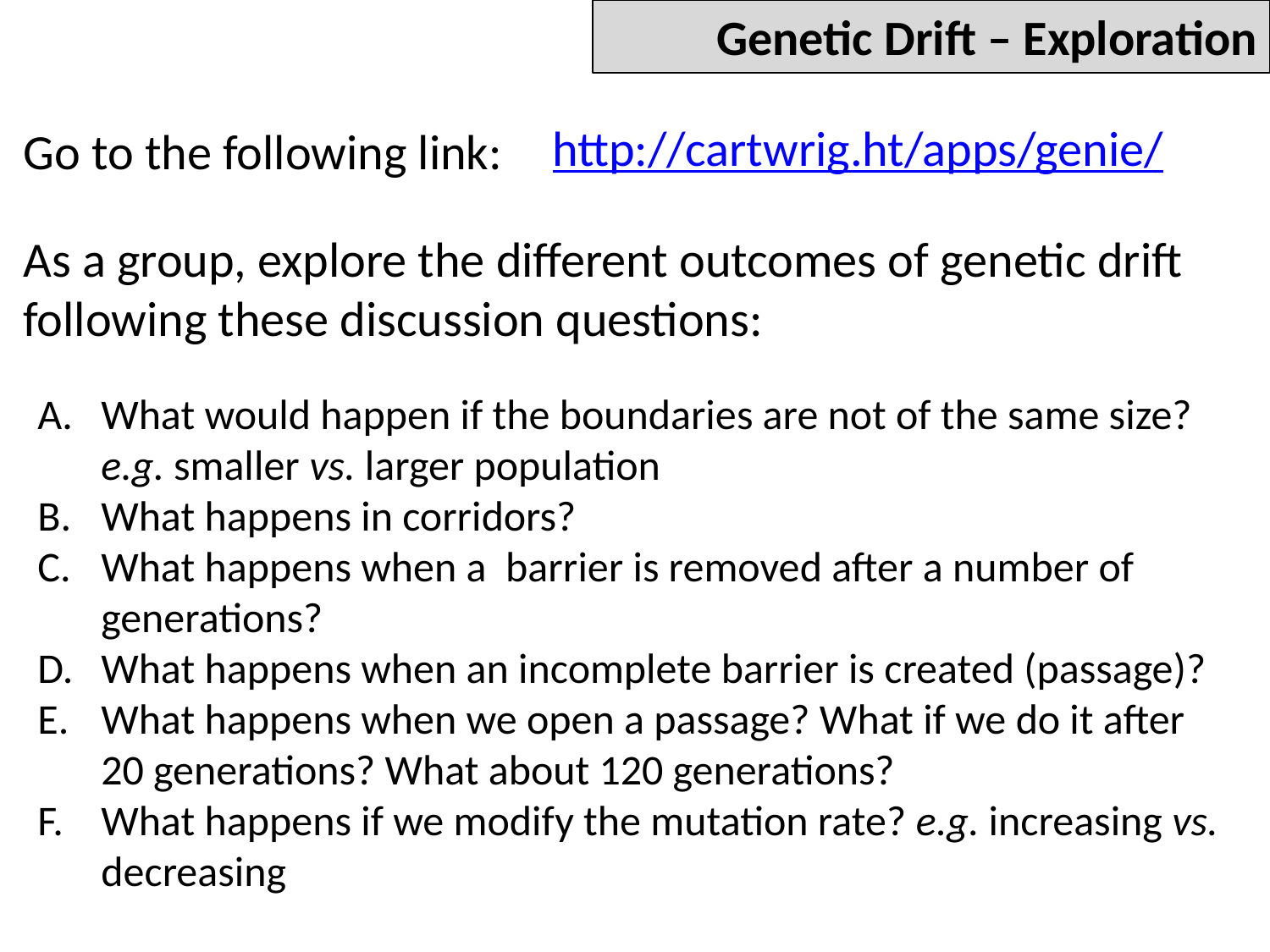

Genetic Drift – Exploration
http://cartwrig.ht/apps/genie/
Go to the following link:
As a group, explore the different outcomes of genetic drift following these discussion questions:
What would happen if the boundaries are not of the same size? e.g. smaller vs. larger population
What happens in corridors?
What happens when a barrier is removed after a number of generations?
What happens when an incomplete barrier is created (passage)?
What happens when we open a passage? What if we do it after 20 generations? What about 120 generations?
What happens if we modify the mutation rate? e.g. increasing vs. decreasing
